# QTL mapping in three tropical maize populations reveals a set of constitutive and adaptive genomic regions for drought tolerance

**DOI:** 10.1007/s00122-012-2003-7

**Published:** 2012-11-04

**Authors:** Gustavo Dias Almeida, Dan Makumbi, Cosmos Magorokosho, Sudha Nair, Aluízio Borém, Jean-Marcel Ribaut, Marianne Bänziger, Boddupalli M. Prasanna, Jose Crossa, Raman Babu

**Affiliations:** 1 Universidade Federal de Viçosa (UFV), CEP 36.570-000 Viçosa, Minas Gerais Brazil; 2 International Maize and Wheat Improvement Center (CIMMYT), Apdo. Postal 6-641, Mexico, DF Mexico; 3 CIMMYT, ICRAF House, United Nations Avenue, Gigiri, Nairobi, 00621 Kenya; 4 CIMMYT, P.O. Box MP 163, Mount Pleasant, Harare Zimbabwe; 5 Generation Challenge Program, hosted By CIMMYT, Apdo. Postal 6-641, Mexico, DF Mexico

## Abstract

Despite numerous published reports of quantitative trait loci (QTL) for drought-related traits, practical applications of such QTL in maize improvement are scarce. Identifying QTL of sizeable effects that express more or less uniformly in diverse genetic backgrounds across contrasting water regimes could significantly complement conventional breeding efforts to improve drought tolerance. We evaluated three tropical bi-parental populations under water-stress (WS) and well-watered (WW) regimes in Mexico, Kenya and Zimbabwe to identify genomic regions responsible for grain yield (GY) and anthesis-silking interval (ASI) across multiple environments and diverse genetic backgrounds. Across the three populations, on average, drought stress reduced GY by more than 50 % and increased ASI by 3.2 days. We identified a total of 83 and 62 QTL through individual environment analyses for GY and ASI, respectively. In each population, most QTL consistently showed up in each water regime. Across the three populations, the phenotypic variance explained by various individual QTL ranged from 2.6 to 17.8 % for GY and 1.7 to 17.8 % for ASI under WS environments and from 5 to 19.5 % for GY under WW environments. Meta-QTL (mQTL) analysis across the three populations and multiple environments identified seven genomic regions for GY and one for ASI, of which six mQTL on chr.1, 4, 5 and 10 for GY were constitutively expressed across WS and WW environments. One mQTL on chr.7 for GY and one on chr.3 for ASI were found to be ‘adaptive’ to WS conditions. High throughput assays were developed for SNPs that delimit the physical intervals of these mQTL. At most of the QTL, almost equal number of favorable alleles was donated by either of the parents within each cross, thereby demonstrating the potential of drought tolerant × drought tolerant crosses to identify QTL under contrasting water regimes.

**Electronic supplementary material:**

The online version of this article (doi:10.1007/s00122-012-2003-7) contains supplementary material, which is available to authorized users.

## Introduction

Drought is one of the most important constraints of global agriculture and severely affects maize, the most important staple food crop in Africa. Three-quarters of the world’s severe droughts over the past 10 years have occurred in Africa, resulting in extreme variation in aggregated regional production, which has ranged from 7.6 to 22.7 million tonnes, and has exhibited close correlation with rainfall (Bänziger et al. 2006). Though drought affects maize at almost all growth stages, the crop is extremely sensitive in the period from 1 week before to 3 weeks after flowering (Bänzinger et al. 2000). Maize is widely regarded to be more susceptible to drought at flowering than other rain-fed crops. This is due to a combination of several factors including physical separation of male and female flowers, floral asynchrony, non-receptivity of the silk, tassel blasting, trapped anthers and embryo abortion (Westgate and Boyer 1985; Lu et al. 2011). Consequently, breeding maize for reproductive-stage drought tolerance could lead to the development of improved varieties that are able to withstand varying degree of water stress (Bolaños and Edmeades 1996; Ribaut et al. 1997; Messmer et al. 2009; Zhu et al. 2011).

Duvick (2005) estimated the rate of breeding progress for temperate maize germplasm under mild drought to be 0.85 % per year for hybrids released between 1930 and 1990, and slightly less under optimal conditions. The significant breeding gain in temperate maize under drought stress has been attributed mainly to the use of rain-fed breeding nurseries with high plant densities and large-scale multi-location testing (Bänziger and Araus 2007). Plant water and nutrient deficits occur more readily under high plant densities and the large-scale multi-location testing frequently exposed newer hybrids to drought conditions (Tsonev et al. 2009). With the introduction of ‘managed stress’ screening, especially for reproductive-stage drought tolerance, a higher breeding progress of 2–2.5 % per year was reported (Campos et al. 2004). Over a shorter breeding history, yield gains of 3.8–6.3 % per year under drought and slightly less under optimal conditions have been reported for tropical maize (Bänziger and Araus 2007). These gains were mainly associated with increased flowering synchronization, fewer barren plants, a smaller tassel size, a greater harvest index, and delayed leaf senescence (Ribaut et al. 2009).

Tolerance to drought in maize is a polygenic trait and typically has low heritability and characterized by high genotype × environment interaction (GEI). Conventional breeding based on direct selection of phenotypes under drought has led to impressive yield gains in maize but underlying genetic causes largely remain unknown. Quantitative trait loci (QTL)-based approaches can contribute significantly to the understanding of genetic basis of crop performance especially under drought stress conditions and such knowledge may be crucial in designing cost-effective breeding approaches aimed at improving sustainability and stability of grain yield under adverse conditions (Collins et al. 2008).

In maize, QTL mapping for grain yield (GY) under water stress and other associated traits such as anthesis-silking interval (ASI) have been an active area of research especially in the past two decades. The QTL detected under water-stress and well-watered (WW) conditions can be categorized according to the stability of their effects across environmental conditions. A ‘constitutive’ QTL is consistently detected across most environments, while an ‘adaptive’ QTL is detected only in specific environment such as WS conditions (Collins et al. 2008). One of the earliest studies involving tropical germplasm under managed stress conditions identified 13 QTL on chromosomes 1, 2, 4, 6, 7, 8 and 10 for grain yield, of which QTL on chromosomes 1 and 10 were stable across WW and WS environments (Ribaut et al. 1997). Since then, a number of QTL regulating morpho-physiological component traits as well as GY have been reported in maize (Malosetti et al. 2008; Messmer et al. 2009, 2011; Li et al. 2010). An updated compilation of mapped QTL and major genes associated with abiotic stress tolerance including drought in maize is available at http://www.maizegdb.org as well as http://www.plantsress.com. Drought tolerance QTL studies in maize and other crops and the strategies for their use in marker-assisted selection (MAS) in breeding programs have been extensively discussed in several comprehensive reviews (Ribaut and Ragot 2007; Araus et al. 2008; Collins et al. 2008; Ribaut et al. 2009; Tuberosa and Salvi 2009).

While genetic dissection of drought tolerance in maize seems to have been widely reported, accounts of successful practical application of identified QTL in maize improvement programs have been scarce. The reasons are manifold, including genetic complexity, influence of genetic background, epistasis, profound QTL × environment interactions (QEI), population-specific nature of identified QTL and involvement of donor lines that are not agronomically elite (Collins et al. 2008; Tsonev et al. 2009; Truntzler et al. 2010; Li et al. 2011). Integrating MAS in conventional breeding for drought-related traits will be successful only when constitutive QTL with effects of considerable size that express across a range of elite germplasm are identified. Meta-QTL (mQTL) analysis provides a means of identifying genomic regions responsible for grain yield under water-stress (WS) as well as well-watered conditions across a range of germplasm (Goffinet and Gerber 2000; Li et al. 2011; Swamy et al. 2011). With the availability of whole genome sequence information in maize (Gore et al. 2009), many SNP markers have been physically anchored and are very useful for linkage mapping and QTL identification in maize, and for comparison of results among studies.

We carried out QTL mapping using SNP markers in three tropical populations, involving elite lines across a wide range of environments under contrasting water regimes. Specifically, the objectives of the present investigation were to (1) identify genomic regions influencing GY and ASI across multiple environments under WS and WW conditions and estimation of their effect sizes; (2) determine the stability of the identified QTL across diverse environments; (3) conduct a meta-analysis across three elite tropical populations to identify common genomic regions for GY and ASI and (4) propose a set of SNP markers that physically delimit the identified mQTL to enable integrating MAS for drought tolerance in the conventional maize improvement programs for the tropics.

## Materials and methods

### Plant materials

We evaluated three tropical maize populations that were developed by the Global Maize Program of CIMMYT. Population 1 (CML444 × MALAWI) consisted of 234 recombinant inbred lines (RILs), developed by single-seed descent method. Population 2 (CML440 × CML504) consisted of 247 F_2:3_ families, obtained from randomly chosen F_2_ plants. Population 3 (CML444 × CML441) consisted of 300 F_2:3_ families, obtained from randomly chosen F_2_ plants. Inbred lines CML444, CML441, CML440 and CML504 were developed by CIMMYT, are adapted to mid-altitude (1,000–1,500 m above sea level) regions of sub-Saharan Africa and are considered to be tolerant to water-limited conditions especially at flowering. Inbred Malawi was developed in Zimbabwe and is considered to be relatively sensitive to water-limited conditions, but has a high yield potential under optimal conditions. CML444 and Malawi are of late maturity [937 male growing degree days (GDD)], CML440 and CML441 mature early (824 and 870 male GDD, respectively) and CML504 is of early to intermediate maturity. CML441 and CML444 belong to CIMMYT heterotic group ‘B’ and hence the segregating families of CML444 × Malawi and CML444 × CML441 were testcrossed to CML312 (‘A’ tester). On the other hand, CML440 and CML504 belong to CIMMYT heterotic group ‘B’ and hence segregating families of CML440 × CML504 were testcrossed to CML395 (‘B’ tester) for phenotypic evaluations.

### Field experiments

The field experiments were conducted in Mexico (Tlatizapán station: 18°N, 99°W, 940 m), Kenya (Kiboko station: 2°9′S, 37°75′E, 975 m) and Zimbabwe (Harare: 17°S, 31°E, 1,468 m and Chiredzi: 21°S, 31°E, 392 m). Detailed characterization of these environments for drought phenotyping has been documented by Masuka et al. (2012). In Tlaltizapán, both WW and WS trials were conducted, whereas only WS trials were conducted in Kiboko. In Zimbabwe, the WW experiments were conducted in Harare and WS experiments were conducted at Chiredzi station. The soils at Tlaltizapan are classified as Vertisol, those at Kiboko are Arenosol, while the soils at Harare and Chriedzi are Alfisol. The trials were conducted in 2010 (both WW and WS) and 2011 (WS) in Mexico, whereas in Kenya and Zimbabwe the trials were conducted in 2010. In Zimbabwe, the trials were planted in May at Chiredzi and in November at Harare. In Kenya, trials were planted in June during the rain-free period. Abbreviations for the well-watered environments were MWW for Mexico, ZWW for Zimbabwe; and for water stress environments were MWS for Mexico in 2010, MWS11 for Mexico in 2011, KWS for Kenya and ZWS for Zimbabwe.

The experimental design was alpha (0, 1) lattice (Patterson and Williams 1976) with one-row plots and two replications at all of the locations. In Mexico, plots were 5 m long with 0.75 m between rows. In Kenya, plots were 4 m long with 0.75 m between rows and 20 cm between plants. In Zimbabwe, the plots were 5 m long with spacing of 75 cm between rows and 25 cm between plants in a row. Plots were planted with two seeds per hill and thinned to one plant per hill 3 weeks after planting resulting in plant populations of approximately 66,667 plants ha^−^1 in Mexico and Kenya and 53,333 plants ha^−1^ in Zimbabwe. Fertilizers, insecticides and herbicides were applied as required and in accordance with local recommendation practices. Drought stress was applied according to the established protocols used by CIMMYT (Bänzinger et al. 2000), which are briefly described as below. In Mexico, water was applied to WS trials through furrow irrigation method at 10-day intervals, until 3 weeks before the expected time of anthesis date (AD) in each population. This stress condition was maintained until 5 weeks after 50 % of the families flowered, when one more irrigation was applied. The WS trials in Zimbabwe and Kenya were irrigated with sprinklers once a week until 6 and 2 weeks before and after flowering, respectively. In WW trials at all the locations, the soil moisture was maintained at about field capacity.

For each plot, anthesis date was recorded as the number of days from sowing until at least 50 % of the plants had released pollen, and siking date (SD) was recorded as the number of days from sowing until silks had emerged on at least 50 % of the plants, and ASI was calculated as the difference between SD and AD) (Bolaños and Edmeades 1996). Mature ears were harvested manually, bagged, air-dried and shelled using an electric shelling device. The total grain yield of each plot was weighed on an electronic balance and converted to GY (t/ha) by dividing the total grain weight per plot by the plot area. If variation in the number of plants per plot was statistically significant (*P* < 0.05), it was considered as a covariate in the statistical model to obtain the adjusted means in t/ha of each genotype.

### Statistical analyses of phenotypic data

Variance components were estimated from the standardized plot raw data by linear mixed model analysis using PROC MIXED of SAS (REML option). For individual and combined analysis across locations, a linear model in alpha-lattice design adjusted by a covariate (AD) was used as described by Messmer et al. (2009). In all cases, AD was used as covariate. Best linear unbiased estimators (BLUEs) were estimated, considering genotypes and the covariate as fixed terms and the rest of the terms as random. For estimating broad-sense heritability, all terms were considered random, except the covariate. Broad-sense heritability was estimated by the formula: $h^{2} = \sigma_{\text{g}}^{2} /(\sigma_{\text{G}}^{2} + \sigma_{\text{GE}}^{2} /l + \sigma^{2} /lr)$, where $\sigma_{\text{G}}^{2}$ is the genotypic variance, $\sigma_{\text{GE}}^{2}$ is the GEI, $\sigma^{2}$ is the error variance, *l* is the number of environments and *r* is the number of replications in each trials. The phenotypic and genotypic correlations among traits were calculated as described by Messmer et al. (2011).

### Genetic maps

Genomic DNA was extracted from young leaves collected in a bulk of 15 plants per family or RIL and according to CIMMYT’s laboratory protocols (CIMMYT-Applied Molecular Genetics Laboratory Protocols 2001). Genotyping was done with selected polymorphic markers for each population, from a set of 1,536 SNPs (Yan et al. 2009). SNP genotyping was performed at Kbioscience, UK using the KASPar chemistry.

**Fig. 1:**
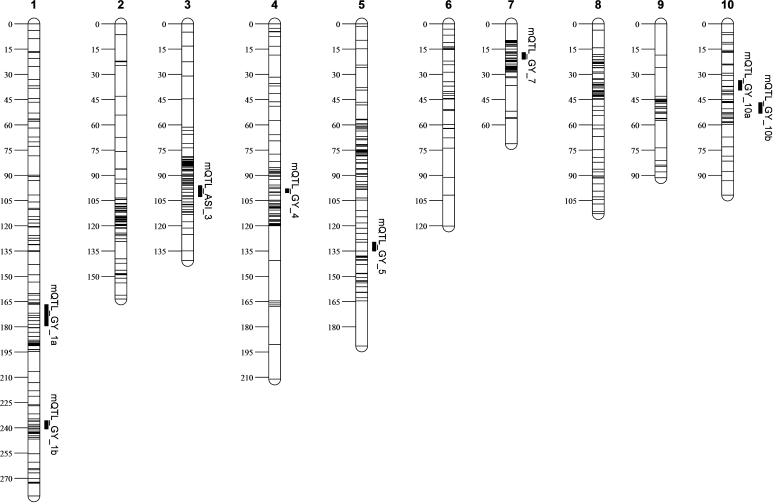
Eight meta-QTL identified based on across population analysis for grain yield (GY) and anthesis-silking interval (ASI). *Short lines* on the consensus chromosome indicate markers positions and *vertical solid bars* to the right of chromosome represent meta-QTL intervals

Linkage maps in all the three populations were constructed using QTL IciMapping ver. 3.2 software (http://www.isbreeding.net) using the twin criterion of more than 3.0 logarithm of odds (LOD) and a maximum distance of 37.2 cM between two loci (Li et al. 2007). Recombination frequencies between linked loci were transformed into cM distances using Kosambi’s mapping function (Kosambi 1944). For CML444 × Malawi, 216 SNP markers were added to an existing linkage map of 160 RFLP and SSR markers (Messmer et al. 2009), providing a total map length of 2,349.2 cM. For the F_2:3_ populations of CML440 × CML504 and CML444 × CML441, linkage maps were constructed using 194 and 265 SNP markers, with 2,712.4 and 3,558.3 cM, respectively. The three distinct genetic maps were merged into a single integrated map using MetaQTL software version 1.0 (Veyrieras et al. 2007). The distances between adjacent markers from all individual maps were rescaled in Haldane units. After integration of all three maps, a consensus map of 620 markers was obtained. The consensus map had a total length of 1,484.5 cM with an average distance of 2.4 cM between markers (Fig. 1).

### QTL analysis

#### Single and multiple environment QTL analysis

QTL were identified for the adjusted means using inclusive composite interval mapping (ICIM) implemented in the software, QTL IciMapping v.3.2 (Li et al. 2007). Three procedures were carried out to identify QTL in each population: (1) mapping QTL for each individual environment, (2) mapping stable QTL across combined WW and combined WS environments within each population and (3) mapping stable QTL across all locations within each population. In all procedures, the walking step in QTL scanning was 1 cM and a relaxed LOD threshold of 2.5 was chosen for declaring putative QTL (Ribaut et al. 1997; Tuberosa et al. 2002). Stable QTL were declared when the LOD of the QEI (LOD_QEI_) was below 2.5. For F_2:3_ populations, additive (a) and dominance (d) effects for each QTL as estimated with QTL IciMapping v.3.2 were used to calculate the ratio of dominance level (|d/a|). This ratio was used to classify the nature of QTL as described by Stuber et al. (1987), which briefly is as follow: additive (A; 0 ≤ |d/a| ≤ 0.2); partially dominant (PD; 0.2 < |d/a| ≤ 0.8); dominant (D; 0.8 < |d/a| ≤ 1.2) and overdominant (OD; |d/a| > 1.2). The sign of the additive effects of each QTL was used to identify the origin of the favorable alleles as proposed by Lubbersted et al. (1997).

#### QTL meta-analysis

QTL meta-analysis was performed with the MetaQTL software version 1.0 (Veyrieras et al. 2007). The statistical method implemented in this software hypothesizes that the input mapping studies are independent from each other. If redundant QTL in the same population in different environments were detected, only the QTL with the highest effect (*R* ^2^) were kept in the analysis. Repeated QTL from the same population but detected in different environments were dropped. A mQTL was declared only when it was shared by all the three bi-parental populations. For a detailed explanation of the methods and procedures adopted in mQTL analysis, see Danan et al. (2011).

## Results

### Phenotypic evaluations across different environments under two water regimes

The estimated means, genetic variance components, heritability and correlation between GY and ASI for the three populations are listed in Table 1. In general, drought stress significantly reduced the GY and increased the ASI across all the environments. In Mexico, between MWW and MWS10, the GY reductions were 41.3, 28.8 and 47.2 % in CML444 × MALAWI, CML440 × CML504 and CML444 × CML441, respectively. The lower GY reduction in MWS10 was due to unexpected rainfall in January (20.0 mm) and February (68.0 mm) of 2010. Across the three populations, combined GY (ALL) means ranged from 1.91 to 9.23 t/ha, whereas GY under stress ranged from 0.1 to 6.76 t/ha. Across all the three populations, strong GEI was observed while no significant negative correlation among locations was noted (Table S1) indicating wide adaptability of these populations across diverse environments.

**Table 1:** Estimates of means, genetic variance components, heritability and phenotypic (*r*_p_) and genotypic (*r*_g_) correlations between grains yield (GY) and anthesis-silking interval (ASI) for families on the three populations in single and multi-environments

Env.	GY (t/ha)								ASI (days)								Correlations	
	Mean	Max	Min	$\sigma_{\text{G}}^{2}$	$\sigma_{\text{GEI}}^{2}$	$\sigma^{2}$	$h^{2}$	CV	Mean	Max	Min	$\sigma_{\text{G}}^{ 2}$	$\sigma_{\text{GEI}}^{2}$	$\sigma^{2}$	$h^{2}$	CV	*r*_p_	*r*_g_
CML444 × MALAWI																		
MWW	9.60	14.69	4.43	1.41	–	2.68	0.51	17.64	0.74	5.31	−1.48	0.25	–	0.78	0.39	127.88	−0.06^ns^	−0.14^ns^
ZWW	7.19	12.33	3.10	0.56	–	3.85	0.22	30.13	0.91	13.50	−1.00	0.05	–	0.67	0.13	91.01	−0.05^ns^	−0.45**
MWS10	5.64	10.50	1.57	1.50	–	1.33	0.74	22.35	2.06	5.99	−2.47	0.65	–	2.29	0.36	74.23	−0.28**	−0.36**
MWS11	4.30	8.15	1.81	0.79	–	2.18	0.63	25.55	4.10	9.53	0.42	1.92	–	3.30	0.54	49.86	−0.51***	−0.87***
KWS	3.83	5.96	2.05	0.36	–	0.46	0.61	19.74	2.02	7.21	0.07	0.83	–	1.34	0.55	60.19	−0.40**	−0.67***
WW	8.58	11.60	5.54	0.14	0.56**	2.96	0.10	19.14	0.87	7.00	−0.75	0.07	0.08^ns^	1.57	0.14	118.11	−0.05^ns^	NA^ф^
WS	4.64	6.76	2.20	0.21	0.91***	1.03	0.31	24.61	2.83	8.34	0.12	0.57	0.68***	2.34	0.48	45.67	−0.53**	NA^ф^
ALL	6.14	8.33	3.68	0.22	0.84***	1.95	0.38	15.99	2.03	5.11	−0.15	0.28	0.52***	2.05	0.48	44.27	−0.21**	NA^ф^
CML440 × CML504																		
MWW	8.61	12.29	4.95	0.86	–	0.77	0.69	10.66	0.85	3.01	−0.50	0.12	–	0.73	0.25	104.06	−0.14*	−0.41*
ZWW	11.39	17.94	6.37	1.47	–	3.95	0.43	18.34	−0.06	2.50	−2.00	0.00	–	1.33	0.00	−1,946.11	−0.10^ns^	NA^ф^
MWS10	6.13	8.23	3.56	0.34	–	0.71	0.49	15.00	2.37	8.33	−0.32	0.39	–	1.77	0.31	59.67	−0.09^ns^	−0.24*
MWS11	4.25	6.59	1.86	0.51	–	0.42	0.71	17.40	4.66	9.89	1.73	1.16	–	2.06	0.53	33.42	−0.53***	−0.86***
KWS	4.24	5.94	2.69	0.11	–	0.47	0.32	17.65	2.50	5.53	0.38	0.42	–	1.04	0.45	42.85	−0.28**	−0.66**
WW	10.00	14.22	6.88	0.61	0.55*	2.40	0.41	13.59	0.40	1.81	−0.79	0.01	0.03^ns^	1.14	0.04	195.24	−0.09*	NA^ф^
WS	4.88	6.36	3.38	0.11	0.22**	0.56	0.39	12.35	3.18	6.15	1.18	0.39	0.26***	1.63	0.52	27.47	−0.26**	−0.65**
ALL	6.93	9.23	5.00	0.23	0.46**	1.31	0.51	9.95	2.07	3.78	0.67	0.18	0.22***	1.41	0.48	30.34	−0.21*	−0.56**
CML444 × CML441																		
MWW	10.81	14.15	2.93	1.73	–	1.33	0.72	10.67	0.35	2.96	−2.03	0.17	–	0.38	0.47	186.81	−0.15*	−0.25*
ZWW	8.99	12.67	2.47	0.96	–	1.75	0.52	14.71	0.33	2.00	−2.00	0.00	–	0.33	0.00	339.80	0.15^ns^	0.07^ns^
MWS10	5.71	9.33	0.74	1.04	–	0.84	0.71	17.56	3.07	7.32	0.45	0.92	–	1.20	0.61	38.17	−0.35***	−0.55***
MWS11	5.33	8.33	2.08	0.71	–	0.68	0.67	17.55	4.70	9.04	0.37	1.14	–	1.74	0.57	30.89	−0.33***	−0.45**
KWS	1.83	3.40	0.09	0.02	–	0.42	0.11	37.15	6.85	15.81	1.51	2.00	–	7.23	0.36	42.73	−0.61**	NA^ф^
ZWS	1.81	3.40	0.39	0.06	–	0.48	0.19	39.59	8.00	16.25	0.66	0.24	–	12.43	0.04	45.27	−0.33***	NA^ф^
WW	9.91	12.66	3.27	0.38	1.05***	1.50	0.30	15.82	0.34	3.00	−2.00	0.09	0.00^ns^	0.76	0.32	218.10	−0.21**	NA^ф^
WS	3.66	5.79	0.10	0.15	0.36***	0.63	0.46	18.25	5.65	11.22	1.48	0.72	0.37***	5.42	0.48	33.34	−0.39***	NA^ф^
ALL	5.73	7.53	1.91	0.30	0.49***	0.91	0.66	11.53	3.88	7.54	−0.29	0.44	0.33***	3.99	0.53	27.03	−0.67***	NA^ф^

*WW* well-watered environments (Mexico and Zimbabwe), *WS* water stress environments (Mexico10, Mexico11, Kenya and ZWS), *ALL* combined analysis across all environments

***, ** and * Significance at *P* < 0.001, 0.01 and 0.05, respectively

Among the three populations, drought stress had the greatest effect in CML444 × CML441, which showed 63 % GY reduction and 94 % increase in ASI, based on combined water stress trials. Among the three populations, heritability for GY ranged from 0.31 to 0.46 under combined WS ($h^{2}_{\text{GYws}}$) and from 0.1 to 0.3 under combined WW environments ($h^{2}_{\text{GYww}}$). Strong GEI for WW locations (as indicated by lower $h^{2}_{\text{GYww}}$) and weak GEI (as indicated by higher $h^{2}_{\text{GYws}}$) in two populations (CML444 × MALAWI and CML444 × CML441) indicates the stability of drought tolerant (DT) genotypes across diverse environments. CML444, which is the common parent shared by these two populations had been previously shown to be stable and high yielding under WS conditions (Messmer et al. 2009). The ranges of ASI values observed within populations were all wider under WS conditions than under WW conditions, and the mean ASI across the three populations was 3.3 days longer under WS conditions than under WW conditions. In all three populations, the genetic variance of ASI was higher in WS environments than in WW environments. Notably, heritability estimates from the combined analysis for WS environments ($h^{2}_{\text{ASIws}}$) were all higher than those from the combined analysis for WW environments ($h^{2}_{\text{ASIww}}$). This reinforces the earlier findings (Messmer et al. 2009; Lu et al. 2011) that reduced ASI is an important common drought adaptive mechanism among different drought tolerant genotypes. Significant and negative phenotypic (*r*_p_) and genotypic (*r*_g_) correlations between GY and ASI were observed across all WS environments across the three populations (Table 1). These correlations were mostly non-significant in WW environments.

### QTL analysis

Single environment QTL analyses revealed 83 and 64 significant QTL for GY and ASI, respectively, among the three populations (Table 2) with varying magnitude of effect sizes. In general, both parents in each of the three populations contributed positive alleles for both traits. Most of the QTL exhibited strong QEI, which was expected keeping in view the diverse environments in Mexico and Africa. In the RIL population of CML444 × MALAWI, fewer QTL were identified because dominant QTL could not be detected. QTL detected in the two F_2:3_ populations across WS and WW environments predominantly showed partial to overdominant effects. In the population CML440 × CML504, around 30 % of the QTL for GY and 15 % for ASI had additive effects (Table S2). The maximum number of QTL was detected in CML444 × CML441, in which only 10 % of the QTL had additive effects for GY and ASI (Table S4).

**Table 2:** Number of QTLs detected by three different mapping procedures in populations CML444 × MALAWI, CML440 × CML504 and CML444 × CML441

Trait	Single environmental QTL						Joint per management		Joint per all env.
	MWW	ZWW	MWS10	MWS11	KWS	ZWS	WW	WS	WW + WS
GY	4/7/7	1/5/2	2/7/9	1/8/7	4/6/7	–/–/6	2/5/2	4/4/1	3/2/0
ASI	0/3/7	0/3/1	3/4/7	2/8/7	5/8/8	–/–/4	1/1/1	5/5/4	4/1/2

In each environment the / separated the population in the followed order (CML444 × MALAWI/CML440 × CML504/CML444 × CML441). The symbol (–) indicates no information in a given environment

### CML444 × MALAWI (tolerant × susceptible)

The single location, individual analyses revealed QTL for GY on almost all the chromosomes, except chromosomes 3 and 6 (Table S2). In the combined analysis across WW environments, one QTL on chromosome 1 (at about 135.0 cM, 101.42–148.69 Mb) had large additive effects (0.62 t/ha in MWS and 0.31 t/ha in ZWW) and explained around 19 % of the phenotypic variance (Table 3). This QTL also consistently showed up in the individual WW analyses (Table S2). The low $R^{2}_{\text{QEI}}$ (2.1 %) indicated the more stable nature of this QTL across WW environments.

**Table 3:** Genetic characteristics of QTLs for GY and ASI mapped across well-watered (WW), water stress (WS) and combined all five environments (ALL) on RILs in population CML444 × MALAWI

Trait	QTL position								Additive genetic effects						Direction
	Env^a^	Chr	Pos. (cM)	Marker interval	Physical position^b^	LOD		*R* ^2^	$R^{2}_{\text{QEI}}$	MWS	ZWW	MWS10	MWS11	KWS	
						G	QEI								
GY	WW	1	135.0	pza02763.1–pza03200.2	101.42–148.69	7.35	0.01	18.93	2.11	0.62	0.31	–	–	–	CML444
	WS	5	225.0	bnlg1346–bnlg118	205.74–211.04	2.92	1.12	4.10	0.63	–	–	0.25	0.10	0.15	CML444
		7	82.0	phm9162.135–bnlg155	137.83–145.24	3.35	0.53	5.43	2.66	–	–	0.33	0.24	0.01	CML444
		9	85.0	umc81–pzb01899.1	49.03–98.50	3.00	0.00	3.49	0.32	–	–	0.17	0.20	0.09	CML444
		10	56.0	pza00337.4–bnlg1079	86.32–89.43	2.83	0.00	2.58	1.77	–	–	0.29	0.03	0.08	MALAWI
	ALL	5	228.0	bnlg118–phm3612.19	216.91–212.48	2.89	1.55	4.42	0.41	0.16	0.13	0.22	0.09	0.21	CML444
		7	82.0	phm9162.135–bnlg155	137.83–145.24	4.02	1.06	5.96	1.90	0.20	0.16	0.33	0.24	0.01	CML444
		9	89.0	umc81–pzb01899.1	49.03–98.50	2.97	0.00	3.19	0.19	0.13	0.11	0.17	0.17	0.09	CML444
ASI	WW	5	142.0	pzb01017.1–pza02641.2	158.03–168.92	2.64	0.00	3.19	0.00	0.10	0.09	–	–	–	MALAWI
	WS	1	139.0	pza02135.2–phm1809.18	166.55–183.29	2.61	0.00	1.62	0.66	–	–	0.11	0.26	0.05	CML444
		2	260.0	pzb00901.4–bnlg2042	9.40–9.54	2.74	0.14	3.08	0.04	–	–	0.22	0.17	0.18	CML444
		3	81.0	pza00210.9–zb21.1	29.69–37.53	2.89	0.00	4.92	0.95	–	–	0.14	0.39	0.19	CML444
		3	175.0	umc3b–umc16a	191.05–205.53	6.66	0.00	8.09	3.20	–	–	0.19	0.59	0.16	MALAWI
		8	55.0	phm2350.17–pza01301.1	23.98–82.23	4.64	0.00	7.03	2.98	–	–	0.05	0.51	0.30	MALAWI
	ALL	1	183.0	pza01039.1–phm3690.23	−217.50**	3.75	0.84	5.06	1.53	0.09	0.13	0.07	0.30	0.27	CML444
		3	137.0	bnl10.24a–PZA02212.1	170.95–174.55	2.53	1.46	4.01	1.68	0.12	0.00	0.24	0.12	0.28	MALAWI
		9	54.0	umc81–pzb01899.1	49.03–98.50	2.79	1.08	2.40	0.70	0.16	0.02	0.15	0.07	0.19	MALAWI
		10	62.0	pza00337.4–bnlg1079	86.32–89.43	2.72	0.00	2.26	0.58	0.03	0.06	0.17	0.16	0.15	CML444

^“**”^Denotes unknown genomic region

^a^Joint analysis across WW: well-watered environments (MWW and ZWW), WS: water stress environments (MWS10, MWS11 and KWS) and combined all five environments (ALL)

^b^Physical positions of the flanking markers on chromosomes in Mb (10^6^ pb). LOD_G_, LOD score for genetic effects across environments; LOD_QEI_, LOD score of the QTL × environment interaction; *R* ^2^, percentage of phenotype variance explained by the QTL across environments; $R^{2}_{\text{QEI}}$, phenotype variation explained by main effects QTL × environment interaction across environments

The combined analysis across WS environments revealed four significant QTL for GY on chromosomes 5, 7, 9 and 10. Interestingly, the QTL, on chromosome 10 had its drought tolerance allele contributed by MALAWI, which is known to be more sensitive to drought stress than CML444 (Messmer et al. 2009). QTL on chromosomes 5, 7 and 9 were also detected across ALL environment analysis, indicating their possible utility in selection decisions across WW and WS environments. Though none of these QTL explained more than 6 % of phenotypic variance for GY, most had very low $R^{2}_{\text{QEI}}$ values indicating their stable nature across diverse environments. Viewed in conjunction with heritabilities for GY (0.31 in WS and 0.38 in ALL), these QTL explain 7–18 % of the genetic variance, which certainly merits their inclusion in marker-based selection indices. The QTL on chromosome 5 was particularly interesting as it had higher additive variance (*R* ^2^ = 4.1) than $R^{2}_{\text{QEI}}$ (0.63), which indicates its consistent performance across WS environments. We detected five significant QTL on chromosomes 1, 2, 3 and 8 for ASI based on the combined analysis of WS environments. Of these, the QTL on chromosome 3 (191.05–205.53 Mb) explained the largest percentage (8 %) of phenotypic variance (Table 3) and was detected in all individual WS environment analyses (Table S2). In contrast to the QTL for GY, most of the QTL detected between WS and ALL environments for ASI were different.

### CML440 × CML504 (tolerant × tolerant)

The individual location QTL analyses for this population revealed 33 and 20 significant QTL for GY and ASI, respectively, spread across all ten maize chromosomes (Table S3). In contrast to what was found in the CML444 × MALAWI (tolerant × susceptible), both parents contributed QTL alleles with positive effects on both GY and ASI. Under WW conditions, the two parents contributed favorable alleles at equal numbers of GY QTL, while CML440 contributed favorable alleles at 66 % of ASI QTL. In WS environments, CML440 contributed favorable alleles at 57 and 60 % of GY QTL and ASI QTL, respectively. The phenotypic variance explained by individual location QTL for GY ranged from 1.5 to 16 % (Table S3).

In the combined analysis across WW environments, we detected five QTL on chromosomes 2, 4, 6, 8 and 9 for GY. Individually, these explained between 11 and 20 % of the phenotypic variance (Table 4). The $R^{2}_{\text{QEI}}$ values for these QTL indicated that all except the one on chromosome 9 were consistent across WW environments. The QTL on chromosome 2 appeared very stable ($R^{2}_{\text{QEI}} = 0.5$) and explained around 20 % of the phenotypic variance for GY. The QTL on chromosome 9 exhibited the largest additive effect: 0.8 t/ha in ZWW, with the favorable allele from CML504. We detected four GY QTL in the combined analysis across three WS environments on chromosomes 1, 5, 7 and 10. Individually these explained between 7 and 17 % of the phenotypic variance. Effect sizes in different WS environments for these GY QTL ranged from 0.02 to 0.18 t/ha. The QTL on chromosome 7, which explained the largest percentage (17 %) of the phenotypic variance for GY in the combined WS analysis, was also detected in all the three individual WS environment analyses (Table S3). At the four GY QTL identified, CML504 contributed the favorable alleles for three of them. The QTL detected on chromosomes 2 and 6 in the WW analysis also showed up in the combined (ALL) analysis, explaining together 37 % of phenotypic variance. We identified four QTL for ASI based on combined analysis across the three WS environments. These were on chromosomes 2, 3, 5 and 9, and individually explained between 8 and 15 % of the phenotypic variance. The ASI QTL on chromosome 5 was in the same position as one detected for GY in the combined WS analysis, providing a partial explanation for the strong genotypic and phenotypic correlation between these two traits, especially under drought stress conditions. The QTL on chromosome 2 explained the largest percentage (15 %) of the phenotypic variance for ASI, performing consistently across the three WS environments ($R^{2}_{\text{QEI}} = 1.9$) with effects ranging from 0.14 to 0.36 days.

**Table 4:** Genetic characteristics of QTLs for GY and ASI mapped across well-watered (WW), water-stress (WS) and combined all five environments (ALL) on F_2:3_ population CML440 × CML504

Trait	Env^a^	Chr	Pos (cM)	Marker interval	Physical position^b^	LOD		*R* ^2^	$R^{2}_{\text{QEI}}$	Additive effects^c^					Direction
						G	QEI			MWW	ZWW	MWS10	MWS11	KWS	
GY	WW	2	118.0	pza01755.1–pza01336.1	25.23–31.39	6.10	0.46	19.43	0.55	0.40^PD^	0.59^PD^	–	–	–	CML440
		4	250.0	phm5599.20–pza03322.5	239.24–242.02	2.52	0.50	11.97	0.44	0.25^PD^	0.30^PD^	–	–	–	CML504
		6	179.0	pzb01222.1–pza02815.25	164.41–167.88	3.32	2.51	13.77	1.38	0.10^OD^	0.27^OD^	–	–	–	CML440
		8	119.0	pza00739.1–pza01049.1	105.79–129.05	2.52	1.25	10.77	1.48	0.19^OD^	0.21^D^	–	–	–	CML440
		9	83.0	pza01791.2–pza000947.1	77.46–96.88	4.06	1.05	12.77	9.57	0.04^OD^	0.79^A^	–	–	–	CML504
	WS	1	262.0	pza03189.4-pza01267.3	64.26–76.05	2.85	2.32	8.40	4.95	–	–	0.18^D^	0.05^OD^	0.17^PD^	CML504
		5	304.0	pza01680.3–pza02480.1	208.90–214.95	4.11	0.86	17.27	8.06	–	–	0.07^OD^	0.05^OD^	0.02^OD^	CML440
		7	134.0	pza03166.1–pza02449.13	137.63–138.55	3.68	0.65	17.75	1.44	–	–	0.12^OD^	0.12^OD^	0.07^OD^	CML504
		10	132.0	pza01141.1–phm3844.14	120.54–146.55	2.81	2.04	6.58	3.61	–	–	0.08^D^	0.10^PD^	0.20^PD^	CML504
	ALL	2	118.0	pza01755.1–pza01336.1	25.23–31.39	8.45	1.38	20.35	13.92	0.37^D^	0.59^PD^	0.09^PD^	0.22^PD^	0.09^PD^	CML440
		6	175.0	pzb01222.1–pza02815.25	164.41–167.88	6.55	9.57	16.92	6.08	0.15^OD^	0.29^OD^	0.14^OD^	0.08^OD^	0.03^D^	CML440
ASI	WW	–	–	–	–	–	–	–	–	–	–	–	–	–	–
	WS	2	111.0	pza01755.1–pza01336.1	25.23–31.39	5.34	2.05	14.77	1.98	–	–	0.32^PD^	0.14^OD^	0.36^PD^	CML440
		3	139.0	phm2290.12–phm15449.10	121.88–125.08	2.72	0.88	8.41	2.93	–	–	0.09^OD^	0.10^OD^	0.14^A^	CML504
		5	305.0	pza01680.3–pza02480.1	208.90–214.95	4.06	0.98	10.08	5.00	–	–	0.13^A^	0.32^D^	0.15^PD^	CML504
		9	120.0	pza01096.1–phm4905.6	133.45–133.88	3.15	0.51	10.23	3.66	–	–	0.32^A^	0.45^A^	0.05^D^	CML504
	ALL	–	–	–	–	–	–	–	–	–	–	–	–	–	–

–, there are no stable QTLs for a give trait across environments

^a^Joint analysis across WW: well-watered environments (MWW and ZWW), WS: water stress environments (MWS10, MWS11 and KWS) and combined all five environments (ALL)

^b^Physical positions of the flanking markers on chromosomes in Mb (10^6^ pb). LOD_G_, LOD score for genetic effects across environments; LOD_QEI_, LOD score of the QTL × environment interaction; *R* ^2^, percentage of phenotype variance explained by the QTL across environments; $R^{2}_{\text{GEI}}$, phenotype variation explained by main effects QTL × environment interaction across environments

^c^The genetic action of the QTLs in each environment determined on the basis of the level of dominance: additive (A), partial dominance (PD), dominance (D) and overdominance (OD)

### CML444 × CML441 (tolerant × tolerant)

We identified a total of 38 and 34 significant QTL for GY and ASI, respectively, based on individual environment QTL analyses (Table S4). The effects of these GY QTL ranged from 0.10 to 0.98 t/ha in WW environments and 0.1 to 0.6 t/ha under WS environments. For ASI QTL identified in various individual environment analyses, the effects ranged from 0.1 to 1.5 days. Favorable alleles at most of the large effect QTL (*R* ^2^ ≥ 10 %) were contributed by CML444 for GY and CML441 for ASI with predominantly additive effects (Table S4). Based on the combined analysis across WW environments, we detected QTL for GY on chromosomes 2 and 3, of which the QTL on chromosome 3 explained 25 % of the phenotypic variance and had effects of 0.9 and 0.1 t/ha in MWW and ZWW, respectively. Favorable alleles for both QTL were contributed by CML444. Most of the identified QTL exhibited overdominant gene action (Table 5).

**Table 5:** Genetic characteristics of QTLs for GY and ASI mapped across well-watered (WW), water stress (WS) and combined all six environments (ALL) on F_2:3_ population CML444 × CML441

Trait	Env^a^	Chr	Pos (cM)	Marker interval	Physical position^b^	LOD		*R* ^2^	$R^{2}_{\text{QEI}}$	Additive effects^c^						Direction
						G	QEI			MWW	ZWW	MWS10	MWS11	KWS	ZWS	
GY	WW	2	34.0	phm4586.12–pza01280.2	30.11–149.43	2.68	1.28	5.31	1.21	0.32^A^	0.18^D^	–	–	–	–	CML444
		3	231.0	pza00279.2–pza02616.1	52.80–210.16	11.19	2.38	24.57	11.48	0.85^PD^	0.10^D^	–	–	–	–	CML444
	WS	3	143.0	pza02098.2–pza03070.9	11.89–43.86	2.77	1.47	9.85	4.30	–	–	0.11^OD^	0.15^OD^	0.09^OD^	0.07^OD^	CML444
	ALL	–	–	–	–	–	–	–	–	–	–	–	–	–	–	–
ASI	WW	1	200.0	phm3034.3–pza01921.19	255.55–261.32	2.60	1.62	14.11	2.68	0.11^PD^	0.11^OD^	–	–	–	–	CML441
		2	125.0	pza01991.3–pza02727.1	220.40–227.92	3.86	0.57	16.65	4.66	–	–	0.13^OD^	0.11^OD^	0.51^OD^	0.46^D^	CML444
		3	140.0	pza02098.2–pza03070.9	11.89–43.86	2.57	0.52	9.55	1.98	–	–	0.07^OD^	0.18^OD^	0.32^D^	0.46^D^	CML441
	WS	6	133.0	phm12794.47–phm1190.3	120.23–128.48	4.37	2.11	14.77	4.70	–	–	0.13^D^	0.36 ^D^	0.43^PD^	0.64^D^	CML444
		7	80.0	pza01909.2–pza01210.1	6.44–75.10	2.81	0.28	5.93	2.05	–	–	0.09^PD^	0.18^A^	0.44^A^	0.48^A^	CML441
	ALL	3	140.0	pza00508.2–pza03070.9	11.89–43.87	3.18	1.18	9.89	4.11	0.09^OD^	0.03^OD^	0.07^OD^	0.14^OD^	0.29^OD^	0.46^D^	CML441
		7	82.0	pza01909.2–pza01210.1	6.44–75.10	3.70	1.36	7.56	4.65	0.16^PD^	0.06^D^	0.09^D^	0.17^A^	0.54^A^	0.48^A^	CML441

–, there are no stable QTLs for a give trait across environments

^a^Joint analysis across WW: well-watered environments (MWW and ZWW), WS: water stress environments (MWS10, MWS11, KWS and ZWS) and combined across all six environments

^b^Physical positions of the flanking markers on chromosomes in Mb (10^6^ pb). LOD_G_, LOD score for genetic effects across environments; LOD_QEI_, LOD score of the QTL × environment interaction; *R* ^*2*^, percentage of phenotype variance explained by the QTL across environments; $R^{2}_{\text{GEI}}$ phenotype variation explained by main effects QTL × environment interaction across environments

^c^The genetic action of the QTLs in each environment determined on the basis of the level of dominance: additive (A), partial dominance (PD), dominance (D) and overdominance (OD)

For ASI, one QTL was identified on chromosome 1 in the combined WW analysis, whereas four QTL were detected in the combined WS analysis, indicating once again the importance of this trait under drought stress conditions. Conspicuously, CML441 contributed favorable alleles at all the identified ASI QTL, which highlighted the potential of this line as a donor for introgression of favorable ASI alleles into other elite germplasm. The QTL on chromosome 2 explained the largest percentage (17 %) of the phenotypic variance, with effects ranging from 0.1 to 0.5 days across various WS environments. The QTL on chromosome 3 appeared most stable (as evidenced by lowest $R^{2}_{\text{QEI}}$ of 1.9), with effects ranging from 0.1 to 0.5 days, and explained close to 10 % of the phenotypic variance. The delimited physical interval for this ASI QTL was the same as that of GY QTL detected on chromosome 3 under WS environments, which again explains the strong correlation between these two traits under drought stress conditions.

When we grouped all six environments in one combined analysis (ALL), no QTL were detected for GY, possibly due to large GEI, indicating relatively narrow adaptation of this population. We identified two QTL on chromosomes 3 and 7 for ASI in the combined analysis (ALL), which together explained 18 % of the phenotypic variance, with effects ranging from 0.1 to 0.5 days (Table 5). The QTL on chromosome 3 was consistently detected for GY as well as ASI across WS environments with significant effects and could be a potential target for marker-assisted breeding programs.

### Meta-analysis

Of the 83 GY QTL identified by single environment analyses (Table 2), we plotted 56 onto a consensus map to perform a mQTL analysis, which enabled a larger overview of genomic regions across the three diverse bi-parental populations. We identified seven mQTL for GY on chromosomes 1, 4, 5, 7 and 10 and one for ASI on chromosome 3 across the three populations with a confidence interval of 95 % (Table 6; Fig. 1). The confidence intervals for the seven mQTL ranged from 2.4 to 12.6 cM, which was well below the arbitrary threshold of 30 cM as established by Hund et al. (2011). The sum of confidence intervals of plotted mQTL covered only 3.2 % (46.93 cM) of the consensus map, built on the three populations. In Table 6, we also provide physical intervals for the mQTL to be able to compare them with previously identified ones and assess their utility in marker-assisted breeding. Except for the mQTL on chromosome 5, which was delimited to an interval of 28 Mb, the mQTL were localized within narrow genomic regions, indicating the efficiency of the analysis. The smallest delimited physical interval corresponded to 2.08 Mb on chromosome 4 (mQTL_GY_4), flanked by PZA03322.5 and PZA01905.12. The mQTL on chromosome 1 (mQTL_GY_1a) had the largest number of QTL integrated, which came from WW as well WS environments. While most of the mQTL for GY included QTL from both WW and WS environments, the mQTL on chromosome 7 (mQTL_GY_7) was based solely on five WS QTL from all the three populations. This region may play an important role in conferring adaptive drought response. As observed earlier, this region was consistently identified across all WS environments in both CML444 × MALAWI and CML440 × CML504 (Tables 3, 4), with low QEI. The favorable alleles at mQTL_GY_7 were contributed by CML444, CML504 and CML441. The mQTL_GY_10 included three WS and two WW QTL, which suggested its possible role in yield stability across both optimal and drought stress conditions. For ASI, only one mQTL was detected on chromosome 3 with an 8.48-Mb physical interval, which included six ASI QTL, all from WS environments, indicating the significance of this genomic region under drought stress conditions.

**Table 6:** Meta-QTLs for GY and ASI across three maize subtropical bi-parental populations identified by meta-analysis

mQTL^a^	Chr	Pos. (cM)	Confidence interval (cM)	Flaking markers	Physical interval (Mb)	QTL number	QTL integrated^b^
mQTL_GY_1a	1	173.17	166.77–179.40	pza02741.1–phm1809.18	161.07–183.29 (22.22)	7	*pop1Gy1_MWW; pop1Gy1_ZWW; pop2Gy1_MWS10; pop2Gy1_KWS; pop3Gy1_ZWW; pop3Gy1a_MWS10; pop3Gy1a_MWS11*
mQTL_GY_1b	1	238.23	235.81–240.65	pza01588.1–pzd1403.1	275.98–285.27 (9.29)	4	*pop1Gy1_MWS11; pop2GY1b_KWS; pop3Gy1b_MWW; pop3_Gy1bMWS10*
mQTL_GY_4	4	99.15	97.95–100.34	pza03322.5–pza01905.12	242.02–244.10 (2.08)	5	*pop1Gy4_KWS; pop2Gy4_KWS; pop2Gy4_MWS11;pop2Gy4_MWW; pop3Gy4_MWS10*
mQTL_GY_5	5	132.33	129.70–134.97	pza00300.14–pza1142.2	171.69–199.70 (28.01)	5	*pop1Gy5_KWS; pop2Gy5_MWS10; pop3Gy5_MWS10; pop3Gy5_MWW; pop3Gy5_ZWS*
mQTL_GY_7	7	19.11	17.66–20.56	pza00986.1–bnl15.21	123.61–132.28 (8.67)	5	*pop1Gy7_MWS10; pop2Gy7_MWS10; pop2Gy7_KWS; pop2Gy7_MWS11; pop3Gy7_ZWS*
mQTL_GY_10a	10	36.48	33.56–39.41	pza00337.4–pza01292.2	86.32–109.63 (23.30)	5	*pop1Gy10_KWS; pop2Gy10_KWS; pop3Gy10_KWS; pop3Gy10_MWS11; pop3Gy10_MWW*
mQTL_GY_10b	10	49.95	46.75–53.18	pza03713.1–phm3736.11	121.49–147.76 (26.27)	5	*pop1Gy10_KWS; pop1Gy10_MWS10; pop2Gy10_MWS10; pop2Gy10_ZWW; pop3Gy10_MWW*
mQTL_ASI_3	3	99.36	96.05–102.67	pzd00027.2–pza01962.1	169.75–178.23 (8.48)	6	*pop1Asi3_KWS; pop1Asi3_MWS11; pop2Asi3_MWS10; pop3Asi3a_KWS; pop3Asi3b_KWS; pop3Asi3_ZWS*

*Mb* megabase (10^6^ pb)

^a^Meta-QTLs (GY for grain yield and ASI for anthesis-silking interval) followed by a chromosome number

^b^Detected QTLs by single QTL analysis in each population among different environments. The three populations were represented by the following order: pop1 (CML444 × Malawi), pop2 (CML440 × CML504) and pop3 (CML444 × CML441)

Using the annotated gene information available in maize database (http://www.maizesequence.org), candidate genes within the mQTL confidence intervals, with possible involvement in GY and ASI under WS and/or WW environments are presented in Table 7. These genes are involved in diverse networks controlling development, metabolism and responses to biotic and abiotic stresses.

**Table 7:** Co-locating candidate genes related to drought tolerance in the physical intervals delimited by meta-QTLs

mQTL	Gene name	Gene position	Gene ID from Gramene	References^a^
mQTL_GY_1a	Cysteine synthase2(*cys2*)	177027403–177032403	GRMZM2G005887	Zhang et al. (2008)
mQTL_GY_1b	Exoglucanase1(*exg1*)	276305014–276310701	GRMZM2G147687	
	Lethal embryo mutant1(*lem1*)	281107355–281109091	AC234157.1_FG002	
	Phosphohexose isomerase1(*phi1*)	283086411–283088116	GRMZM2G065083	
	Aldehyde oxidade (*ZmAO3*)	285274032	GRMZM2G124260	Setter et al. (2011)
mQTL_ASI_3	MADS-domain transcription factor (*Zmm16*)	171427820–171430412	GRMZM2G110153	Whipple et al. (1997), Dwivedi et al. (2008), Setter et al. (2011)
mQTL_GY_5	petD	209941448–209965363	GRMZM2G427444	Raab et al. (2006)
	Glutathione transferase24(*gst24*)	211038250–211039523	GRMZM2G032856	Darko et al. (2011), Chen et al. (2012), Varga et al. (2012)
mQTL_GY_7	Glutathione transferase23(*gst23*)	128373591–128375197	GRMZM2G416632	Marrs (1996), Darko et al. (2011), Chen et al. (2012), Varga et al. (2012)
	Isoamylase-type starch debranching(*iso3*)	129101506–129113096	GRMZM2G150796	
	Glutathione *S*-transferase2(gst2)	90200517–90201913	GRMZM2G132093	Marrs (1996), Darko et al. (2011), Chen et al. (2012), Varga et al. (2012)
mQTL_GY_10a	Cytochrome B6-F complex subunit 5 (*petG*)	90315936–90316307	GRMZM2G547408	Hu et al. (2010)
	NADH dehydrogenase F(*ndhF*)	90140275–90144126	GRMZM2G405584	Casagrande et al. (2001), Pastore et al. (2007)
mQTL_GY_10b	Lipoxygenase7(*lox7*)	120216863–120221081	GRMZM2G070092	Gigon et al. (2004), Peng et al. (2011)
	Glutamine synthetase1 (*gln1*)	146465615–146471079	GRMZM2G098290	Martin et al. (2006), Swarbreck et al. (2011), Yu et al. (2012)
	Transcription factor (*myb2*)	140048665–140050182	GRMZM2G081557	Cominelli et al. (2005), Dubos et al. 2010

^a^Studies reporting the active involvement of these genes with drought tolerance in maize or other species. Authors not listed in the reference section could be found in the supplementary reference

## Discussion

### Stress levels, heritability estimates and correlations between GY and ASI

Ensuring an optimal drought stress is very critical for consistent detection of QTL. Severe water stress conditions reduce genetic variance, adversely affecting the chances of QTL detection (Ribaut et al. 1997). In this investigation, one of the WS environments, MWS10, experienced only moderate drought stress and exhibited higher genetic variance for GY than other WS environments. Reduced genetic variance and heritability estimates for grain yield under severe water stress conditions were also reported by Ribaut et al. (1997); Tuberosa et al. (2002); Messmer et al. (2009) and Lu et al. (2011). In contrast, drought stress always increased genetic variance for ASI: more severe the stress, higher the ASI, which indicates the drought adaptive nature of this trait. In two of the three populations, the heritability estimates for GY in WW environments were considerably less than in corresponding WS environments, indicating significant GEI, which is not surprising considering the diverse nature of environments. However, higher heritability estimates for GY under combined WS environments imply stability of drought tolerant genotypes across diverse environments.

Significant genotypic and phenotypic correlations were observed between ASI and GY, especially in WS environments. These relationships were similar to those already demonstrated in tropical maize germplasm (Bolaños and Edmeades 1996; Ribaut et al. 1997; Malosetti et al. 2008; Messmer et al. 2009; Lu et al. 2011). The strong correlation between GY and ASI is explained in part by the co-location of QTL on chromosomes 3 (11.9–43.9 Mb) and 5 (208.9 to 214.9 Mb) in populations CML440 × CML504 and CML444 × CML441 in WS environments. Other co-locating QTL were identified for GY and ASI on chromosomes 1 and 10 based on single environment analyses. Similar overlapping genomic regions for GY and ASI on chromosomes 1 and 10 were also reported by Ribaut et al. (1997) and Malosetti et al. (2008). This helps explain the strong correlation of ASI with GY across a broad range of germplasm. Higher heritability was recorded for ASI than for GY across all the three populations in WS environments. This suggests that understanding the genetic basis of ASI, which is significantly correlated with GY but has higher heritability, will aid in designing efficient marker-based breeding strategies for enhanced GY under stress conditions.

About 75 % of the yield improvement in maize since 1930s has been attributed to genetic gain and the rest to agronomic practices (Araus et al. 2011). A substantial portion of this genetic gain was not associated with an increase in heterosis but rather with improved stress tolerance (Duvick 2005). Since the discovery of molecular markers, a number of QTL influencing GY under stress and optimal water conditions in maize have been reported (Ribaut et al. 1997; Tuberosa et al. 2002; Lima et al. 2006; Guo et al. 2008; Malosetti et al. 2008; Messmer et al. 2009; Peng et al. 2011; Zhu et al. 2011), but information on QTL that are stable across diverse environments and express more or less uniformly in different genetic backgrounds has been scarce (Li et al. 2010). The location-specific and population-specific nature of QTL, coupled with inconsistent effect sizes, has been a major bottleneck for their utilization in the breeding programs. Here, we report a set of constitutive and adaptive mQTL for GY and ASI that were identified based on three biparental populations, derived from elite tropical germplasm available at CIMMYT. Most of these mQTL had moderate effect sizes for GY and ASI across both WS and WW environments and were delimited to short physical intervals, thereby potentially enabling marker-assisted breeding applications in future.

### QTL analysis across environments and mQTL

In the present investigation, different sets of QTL were identified across different water regimes, but QTL identified in a given WS or WW environment were stable across environments. In two of the three populations, average $R^{2}_{\text{QEI}}$, which is a measure of stability of a QTL, for WS QTL was considerably less than average $R^{2}_{\text{QEI}}$ for WW QTL, indicating the stable nature of adaptive drought tolerant QTL. These results are consistent with findings from other studies, which revealed that GY under WS and WW conditions is controlled by different set of genes (Ribaut et al. 1997; Lu et al. 2011; Messmer et al. 2009) and that a substantial proportion of QTL detected under water stress did not present significant QEIs (Messmer et al. 2009). We used an integrated map of SNPs and SSRs for CML444 × MALAWI population and employed ICIM. This seemed to improve the QTL detection power. For instance, Messmer et al. (2009), who using 160 SSR and RFLP markers and composite interval mapping with one of the same populations, detected QTL for GY under two water regimes on chromosomes 1, 5 and 8, but none of these were in common between locations in Mexico and Africa. Here, we were able to detect a QTL on chromosome 10 (86.32–89.43 Mb) in CML444 × MALAWI, which was expressed in both Mexico and Kenya. This could be due to higher density of markers in the integrated map and/or to improved QTL detection methodology. Though a number of QTL were identified in single environment QTL analyses in the current study as well as previous investigations, many of them were not stable across environments. However, a significant number of QTL were commonly detected either in the same positions or over lapping physical intervals across three different populations, which prompted us to run meta-analysis using all the different QTL identified in single environment analyses. We present here seven mQTL regions for GY and one for ASI that were identified based on their expression in all the three populations, either in one or more environments under two water regimes. Of the seven mQTL for GY, except the one on chr.7, all other mQTL integrated QTL from WS as well as WW conditions. Particularly, mQTL_GY_1a and mQTL_GY_10b integrated almost equal number of QTL under WS and WW environments, suggesting the significant role of these genomic regions under both stress and optimal conditions. The mQTL on chr.7 (mQTL_GY_7) solely integrated QTL from WS environments, indicating the adaptive nature of this region. The rest of the regions were predominantly indicated by WS QTL while integrating at least one WW QTL.

### Constitutive mQTL regions for GY

In the same physical interval as that of the constitutive mQTL_GY_1a (1.05/0.06 at 161.07–183.29 Mb), a number of studies earlier have reported QTL for GY and ASI, implying the significance of this region not only for WS conditions but also for optimal environments. Using RFLP markers in a F_3_ population of tropical maize, Ribaut et al. (1997) identified a QTL on 1.06 for GY across WW and WS environments. Tuberosa et al. (2002) reported a SSR, *csu61b*, which is located between 180.71 and 181.19 Mb on chromosome 1 to be strongly linked with GY and root traits under both stress and optimal water conditions. More recently, Messmer et al. (2009) evaluating the RILs of CML444 × Malawi identified a cluster of QTL on bin 1.06 related to GY and other yield contributing traits under drought as well as WW conditions in Mexican and African environments. A stable QTL for GY under WW conditions based on five Brazilian environments was detected in the physical interval of 91.46–185.02 Mb on chrmosome1 in yellow tropical maize germplasm (Lima et al. 2006). Similarly, Lu et al. (2010) using a F_2:3_ population, identified a QTL in bin 1.06 (164.55–195.05 Mb) for GY under WW conditions based on means across seven Asian environments. A recent meta-analysis involving 17 independent QTL mapping studies detected 3 strong genomic regions on chromosomes 1, 7 and 10, of which the mQTL region on chromosome 1 was delimited to the physical interval, between 178.87 and 180.72 Mb in bins 1.05 and 1.06 (Li et al. 2010), reinforcing the evidence for the constitutive effect of this genomic region.

Another region for which strong evidence of constitutive effects on GY is on chromosome 10 (mQTL_GY_10b) in the physical interval from 121.49 to 147.74 Mb (bins 10.04–10.07). Upstream of this region, another mQTL (mQTL_GY_10a) was identified at bin 10.04 at about 86.33- to 109.63-Mb interval, which, however, was more prevalent in WS environments. The mQTL_GY_10b genomic region was also identified as stable across WS environments in CML440 × CML504, while mQTL_GY_10a was identified as stable in CML444 × MALAWI. These regions have also been detected in previous studies (Ribaut et al. 1997; Guo et al. 2008; Malosetti et al. 2008; Hao et al. 2010, 2011; Li et al. 2010; Peng et al. 2011; Setter et al. 2011), which reported a number of GY and ASI QTL under drought as well as WW conditions across diverse maize germplasm. In a mQTL analysis that integrated results from 12 QTL mapping experiments, Hao et al. (2010) identified a significant genomic region for GY under WS conditions in bin 10.04 and another constitutive region for GY in bin 10.06. Another mQTL analysis by Li et al. (2010) involving seven other populations detected four important regions on chromosome 10 for GY and related traits under drought conditions. All of these overlapped with the intervals delimited in the present investigation. Guo et al. (2008) reported a QTL for drought tolerance index on chromosome 10 between 141.86 and 146.06 Mb, whereas Peng et al. (2011) identified a QTL for GY under combined WW conditions between 111.84 and 126.62 Mb. Interestingly, using a mixed model approach, Malosetti et al. (2008) detected a strong QTL for drought as well as low nitrogen tolerance at 124.32 Mb. Taken together, all these results imply that the genomic regions identified on chromosome 10 may play important roles in conferring yield advantages not only under drought stress but also in optimal environments. However, unless resolved through further fine mapping studies, it would be difficult to conclude whether there are many QTL clustered in these regions or whether pleiotropy is responsible for the manifold effects identified in the current and previous studies. Bioinformatic analysis of the physical interval (121.49–147.74 Mb) on chromosome 10 delimited to mQTL_GY_10b, based on the ‘Named Genes’ annotation track (http://www.plantgdb.org/ZmGDB) revealed the three important candidate genes that have been previously linked to GY under drought and/or optimal conditions either in maize or other species: lipoxygenase7 (*lox7*) (GRMZM2G070092), glutamine synthetase1 (*gln1*) (GRMZM2G098290) and *Myb2* transcription factor (GRMZM2G081557) (Table 7). Lipoxygenases have been reported to respond to biotic stresses and to certain abiotic conditions such as water deficit and wounding (Bell and Mullet 1991). Glutamine synthetase (GS) is important for nitrogen flow in plant metabolism and has been strongly implicated in maize grain production (Martin et al. 2006; Swarbreck et al. 2011), with overexpression of GS1-3 resulting in 30 % increase in kernel number. Interestingly, Medici et al. (2003) showed that GS activity is not affected by drought in maize hybrids subjected to severe water stress, which tempts us to speculate that *gln1* may be a candidate for one of the QTL identified under WW conditions in this region. *Myb2* transcription factor has been shown to be an important transcriptional modulator of physiological responses in guard cells through a null mutation in *AtMyb60*, which resulted in the constitutive reduction of stomatal opening and in decreased wilting under water stress conditions (Cominelli et al. 2005 and Dubos et al. 2010).

Chromosome 1 harbored another mQTL genomic region downstream to the one described earlier, at 275.98–285.27 Mb, which predominantly integrated QTL for GY under WS conditions and one for GY under WW environment. Using a F_2:3_ population of Qi319 × Huangzaosi and SSR markers, Peng et al. (2011) identified a stable QTL in the interval between 258.88 and 292.98 Mb interval, based on across WW environments. Unlike mQTL_GY_1a, this region has not been reported by many earlier studies that used biparental populations. However, with an association mapping approach and a set of 1,229 SNPs in a panel of about 350 inbred lines from CIMMYT, which included 5 parental lines used in the current study, Setter et al. (2011) detected a significant association between a SNP marker (PZB01403.4) at 285.27 Mb and to the abscisic acid (ABA) levels in silks 7 days after flowering under water stress condition across 2 years in Mexico. The SNP is located within a gene (GRMZM2G124260) with aldehyde oxidase activity that is known to catalyze a wide range of reactions, including ABA synthesis (Ibdah et al. 2009). Abscisic acid is a fundamental component of the complex mechanisms that allow the plant to match water supply with the water demand. This hormone has been shown to affect many traits influencing the water balance of the plant through mechanisms of dehydration avoidance and dehydration tolerance (Giuliani et al. 2005). Another genome-wide association study using a 1,536 SNP chip and set of 95 inbred lines that are parents of popular hybrids in China identified three SNPs on chromosome 10 (PZB02529.1 at 86.32 Mb, PZB0111.8 at134.03 Mb and PZA03607.2 at 141.82 Mb) as strongly associated with GY, ASI and drought tolerance index across different environments (Hao et al. (2011). Setter et al. (2011), using a panel of inbred lines mentioned above, identified a region associated with accumulation of phasic acid in maize ears on chromosome 10 at 138.76 Mb. The SNP in this region is located within an aquaporin gene (GRMZM2G125023) that is known to be essential for regulation of water movement in cells (Devis et al. 2012).

Two other constitutive genomic regions detected as mQTL in the present investigation were on chromosomes 4 and 5. The mQTL_GY_4 which integrated 4 QTL from WS and one from WW environment seems to be novel, as there are no previous reports of similar QTL in this region. The mQTL on chromosome 5 is in a region (171.69–199.70 Mb, bin 5.05/06/07) in which QTL have been detected in other studies (Messmer et al. 2009; Hao et al. 2010; Li et al. 2010) that evaluated either lines or hybrids under drought and/or WW conditions. Particularly, it is worth mentioning that the meta-analyses conducted by Hao et al. (2010) and Li et al. (2010) identified a constitutive QTL in bin 5.06 and an adaptive region in bin 5.07, thereby providing strong evidence for this delimited physical interval.

### Stress-adaptive mQTL regions for GY and ASI

Unlike other mQTL for GY that integrated at least one QTL under WW condition, the mQTL in bin 7.03 at 122.62–132.28 Mb interval integrated only QTL from WS environments and hence seemed adaptive in nature. This region was also found to be stable across all WS environments in CML444 × MALAWI as well as CML440 × CML504. The adaptive nature of this region is strongly supported by the meta-analysis of Li et al. (2010) and the association study of Hao et al. (2011), which reported two significant markers at 122.62 Mb and 133.37 Mb in bin 7.03 associated with GY, ASI and drought tolerance index, only under WS conditions.

Physical intervals delimited to mQTL on chromosomes 5 and 7 harbored genes belonging to glutathione *S*-transferases (GST) family (*gst24*, *gst23* and *gst2*, Table 7). In plants, glutathione *S*-transferases are known to play significant regulatory roles and are induced by diverse environmental stimuli such as dehydration, senescence, and wounding with increased GST levels used to maintain cell redox homeostasis and protect organisms against oxidative stress. GSTs have been proposed to afford protection against various stress conditions by detoxifying endogenous plant toxins that accumulate as a consequence of increased oxidative stress (Marrs 1996). Recently, enhanced activity of GSTs under water stress conditions was reported to confer selective advantages in maize (Darko et al. 2011) and in winter wheat (Varga et al. 2012). Using a knock-out mutant for a GST gene in *Arabidopsis*, Chen et al. (2012) demonstrated that GSTs play a pivotal negative regulatory role in conferring drought and salinity tolerance to the mutant plants as compared to wild ones.

Similar to GY mQTL in bin 7.03, the mQTL_ASI_3 was detected only across WS environments, which is consistent with the individual QTL analysis results wherein ASI was found to be relevant only under drought conditions. The physical interval delimited to mQTL_ASI_3 (96.05–02.67 Mb) contains a candidate gene, *Zmm16* (GRMZM2G110153—MADS-domain transcription factor) that has been clearly implicated in reproductive organ development (Whipple et al. 1997; Dwivedi et al. 2008; Setter et al. 2011).

Most of the previous studies detected QTL for drought tolerance based on populations derived from crosses between tolerant and susceptible parents. In this investigation, a number of QTL for GY and ASI were detected in populations derived from crosses between two tolerant parents that have immediate relevance to practical breeding programs. CML444 was involved in two crosses and the favorable alleles contributed by this genotype at different mQTL were consistent. It is likely that the favorable haplotypes at different coincident QTL across three populations detected by mQTL analysis were the same, which, however, can only be validated with further fine mapping experiments.

In the tropics, rain-fed maize cultivation is often exposed to extended periods of water limitation, both during vegetative and reproductive phases, which necessitates selection for stable GY especially under WS conditions. At the same time, efforts to impart drought tolerance should not result in compromised GY under optimal conditions. This requires identification of genotypes that equally perform well under WS and WW conditions. In the present study, we have identified several families within three subtropical biparental populations that combine high GY under WW environments with good tolerance to WS conditions (Table S5), which could serve as an excellent source of initial source population for marker-assisted recurrent selection in tropical breeding programs. Seeds of these superior families can be requested from CIMMYT by contacting the corresponding author. Although the genetics of GY differ considerably between WW and WS conditions, this study has demonstrated that it is possible to identify genomic regions that confer selective advantages under WS, without compromising the optimal GY potential. The eight mQTL regions identified in the present investigation merit attention for use in the MAS as well as marker-assisted recurrent selection activities within pedigree breeding and population improvement programs.

## Electronic supplementary material

Below is the link to the electronic supplementary material.

Supplementary material 1 (DOCX 88 kb)
