# Supplementary material for: QTL mapping in three tropical maize populations reveals a set of constitutive and adaptive genomic regions for drought tolerance
Source: Theor Appl Genet. 2012 Nov 4;126(3):583–600. doi: 10.1007/s00122-012-2003-7 (PMC3579412; doi:10.1007/s00122-012-2003-7)
Supplement: Supplementary file 1 — Supplementary material 1 (DOCX 88 kb) [file 122_2012_2003_MOESM1_ESM.docx]

**Supplementary:**

**Table S1.** Phenotypic (below diagonal) and genotypic (above diagonal) correlations of environments based on three subtropical maize populations for GY

|  | | **Population CML444xMALAWI** | | | | | |
| --- | --- | --- | --- | --- | --- | --- | --- |
| Envir. | MWW | | ZWW | MWS10 | MWS11 | KWS | ZWS |
| MWW | 1 | | 0.14 ^ns^ | 0.31^**^ | 0.32^*^ | 0.20 ^ns^ | - |
| ZWW | 0.05 ^ns^ | | 1 | 0.30^**^ | -0.24 ^ns^ | 0.12 ^ns^ | - |
| MWS10 | 0.17^*^ | | 0.10 ^ns^ | 1 | 0.24 ^ns^ | 0.30^**^ | - |
| MWS11 | 0.19^**^ | | -0.09 ^ns^ | 0.13 ^ns^ | 1 | 0.41^***^ | - |
| KWS | 0.14 ^ns^ | | 0.05 ^ns^ | 0.10 ^ns^ | 0.27^***^ | 1 | - |
| ZWS | - | | - | - | - | - | - |
|  | | **Population CML440x** **CML504** | | | | | |
| MWW | 1 | | 0.54^***^ | 0.39^**^ | 0.22 ^ns^ | 0.42^***^ | - |
| ZWW | 0.29^***^ | | 1 | 0.37^**^ | 0.02 ^ns^ | 0.05 ^ns^ | - |
| MWS10 | 0.22^**^ | | 0.17^*^ | 1 | 0.21 ^ns^ | 0.16 ^ns^ | - |
| MWS11 | 0.15^*^ | | 0.01 ^ns^ | 0.13 ^ns^ | 1 | 0.24 ^ns^ | - |
| KWS | 0.20^**^ | | 0.02 ^ns^ | 0.06 ^ns^ | 0.11 ^ns^ | 1 | - |
| ZWS | - | | - | - | - | - | - |
|  | | **Population CML444xCML441** | | | | | |
| MWW | 1 | | 0.27 ^ns^ | 0.63^***^ | 0.57^***^ | -0.03 ^ns^ | 0.44^**^ |
| ZWW | 0.17^**^ | | 1 | 0.25 ^ns^ | 0.31^**^ | 0.15 ^ns^ | 0.32^**^ |
| MWS10 | 0.45^***^ | | 0.16 ^ns^ | 1 | 0.46^***^ | -0.13 ^ns^ | 0.37^**^ |
| MWS11 | 0.40^***^ | | 0.19^**^ | 0.32^***^ | 1 | 0.00 ^ns^ | 0.28 ^ns^ |
| KWS | -0.02 ^ns^ | | 0.09 ^ns^ | -0.04 ^ns^ | 0.00 ^ns^ | 1 | 0.49^***^ |
| ZWS | 0.17^**^ | | 0.11 ^ns^ | 0.14 ^ns^ | 0.11 ^ns^ | 0.18^**^ | 1 |

Numbers followed by ^***^,^**^, ^*^ and ^ns^ indicate significance at P < 0.001, 0.01, 0.05 and no-significant effect, respectively. (-) environments removed from the analysis due to very low genetic variance under drought stress.

**Table S2.** QTLs and their genomic position, LOD values, genetic effects, gene action and phenotypic variation explained (R^2^) for GY and ASI QTLs mapped in different environments using RILs families of CML444xMALAWI

| **Env.** | **QTL**^1^ | **Chr** | **Pos (cM)** | **Marker Interval** | **Physical position**^2^ | **LOD** | **R^2^ (%)** | **Add**^3^ | **Direction** |
| --- | --- | --- | --- | --- | --- | --- | --- | --- | --- |
| MWW | *Gy1* | 1 | 153.0 | csu61b-bnlg1057 | 190.95-191.0 | 3.93 | 7.52 | 0.43 | CML444 |
|  | *Gy2* | 2 | 239.0 | phm12979.9-pzao2337.4 | 9.03-15.51 | 3.19 | 6.43 | -0.39 | MALAWI |
|  | *Gy7* | 7 | 113.0 | bnl14.07-pza00795.1 | 159.41-162.15 | 6.41 | 12.29 | 0.55 | CML444 |
|  | *Gy9* | 9 | 121.0 | umc105a-umc11a | 17.81-34.66 | 4.56 | 12.27 | 0.54 | CML444 |
|  | *Asi* | - | - | - | - | - | - | - | - |
|  |  |  |  |  |  |  |  |  |  |
| ZWW | Gy1 | 1 | 130.00 | bnlg2086-phm10621.29 | 82.34-101.42 | 3.17 | 11.04 | 0.52 | CML444 |
|  | Asi | - | - | - | - | - | - | - | - |
|  |  |  |  |  |  |  |  |  |  |
| MWS10 | *Gy7* | 7 | 77.0 | phm3078.12-phi082 | 128.47-131.39 | 2.57 | 6.59 | 0.38 | CML444 |
|  | *Gy10* | 10 | 47.0 | phm15331.16-pza01597.1 | 10.43 - 61.60 | 4.36 | 17.57 | -0.62 | MALAWI |
|  | *Asi3* | 3 | 123.0 | pza00186.4-pzad00027.2 | 165.80 - 169.75 | 2.88 | 9.31 | 0.41 | CML444 |
|  | *Asi4* | 4 | 95.0 | umc156a-pza00453.2 | 143.10 -166.28 | 2.74 | 11.56 | 0.46 | CML444 |
|  | *Asi10* | 10 | 126.0 | bnlg236-umc1038 | 140.96- 148.09 | 3.35 | 10.67 | 0.44 | CML444 |
|  |  |  |  |  |  |  |  |  |  |
| MWS11 | Gy1 | 1 | 264.0 | bnlg1720-d8.3 | 264.72-265.19 | 2.85 | 6.68 | 0.31 | CML444 |
|  |  |  |  |  |  |  |  |  |  |
|  | Asi3 | 3 | 173.0 | umc7-umc3b | 165.99-196.07 | 4.9 | 10.34 | -0.65 | MALAWI |
|  | Asi5 | 5 | 48.0 | umc147a-pza02462.1 | 5.48-6.82 | 3.84 | 8.78 | 0.59 | CML444 |
|  |  |  |  |  |  |  |  |  |  |
| KWS | *Gy4* | 4 | 101.0 | pza00453.2-bnl2291 | 166.28-168.61 | 6.57 | 19.38 | 0.33 | CML444 |
|  | *Gy5* | 5 | 230.0 | bnlg118-phm3612.19 | 211.04-212.48 | 3.55 | 10.98 | 0.25 | CML444 |
|  | *Gy8* | 8 | 140.0 | pza01964.29-umc1384 | 166.98-168.36 | 6.16 | 18.49 | 0.33 | CML444 |
|  | *Gy10* | 10 | 32.2 | npi285a-pza00048.1 | 5.45- 98.58 | 3.20 | 7.24 | -0.20 | MALAWI |
|  |  |  |  |  |  |  |  |  |  |
|  | *Asi1* | 1 | 283.0 | phm13362.3-umc147b | 275.98- 281.86 | 3.74 | 7.75 | -0.34 | MALAWI |
|  | *Asi2* | 2 | 83.0 | pza01352.5-pza01991.3 | 220.39-226.45 | 6.12 | 15.18 | -0.48 | MALAWI |
|  | *Asi3* | 3 | 149.0 | pza02212.1-phm17210.5 | 174.55-178.22 | 4.57 | 10.64 | -0.40 | MALAWI |
|  | *Asi4* | 4 | 77.0 | pza03409.1-csu100 | 128.63-136.06 | 3.22 | 8.18 | -0.35 | MALAWI |
|  | *Asi10* | 10 | 115.0 | bnlg236-pza00130.9 | 140.96-143.26 | 3.06 | 7.44 | -0.33 | MALAWI |
|  |  |  |  |  |  |  |  |  |  |

^1^Name of QTLs (*Gy* for grain yield and *Asi* for anthesis-silking interval) followed by the chromosome number. ^2^Physical position of flanking marker of the QTL in Mb (10^6^ bp). ^3^QTLs with additive effects with positive values were contributed by the parent CML444 and QTL with negative values are from parent MALAWI.

**Table S3.** QTLs and their genomic position, LOD values, genetic effects, gene action and phenotypic variation explained (R^2^) for GY and ASI QTLs mapped in different environments using F_2:3_ families from CML440xCML504

| **Env.** | **QTL Position** | | | | | **LOD** | **Genetic Effect**^3^ | | | **Gene Action**^4^ | |  |
| --- | --- | --- | --- | --- | --- | --- | --- | --- | --- | --- | --- | --- |
|  | **QTL**^1^ | **Chr** | **Pos (cM)** | **Marker Interval** | **Physical positon** ^2^ |  | **R^2^ (%)** | **Add** | **Dom** | **\|d/a\|** | **Nature** | **Direction** |
| MWW | *Gy2* | 2 | 121.0 | pza01755.1-pza01336.1 | 25.23-31.39 | 3.60 | 7.77 | 0.40 | -0.40 | 1.00 | D | CML440 |
|  | *Gy3* | 3 | 304.0 | pza01688.3-phm2423.33 | 223.67 - 227.68 | 2.81 | 6.70 | -0.35 | -0.37 | 1.06 | D | CML504 |
|  | *Gy4* | 4 | 228.0 | phm5599.20-pza03322.5 | 239.23 - 242.02 | 3.90 | 3.54 | -0.27 | -0.25 | 0.95 | D | CML504 |
|  | *Gy5* | 5 | 155.0 | pza00996.1-pza01530.1 | 37.78 -37.79 | 3.67 | 7.49 | 0.46 | 0.12 | 0.27 | A | CML440 |
|  | *Gy6* | 6 | 172.0 | pzb01222.1-pza02815.25 | 164.41 - 167.88 | 4.64 | 8.81 | -0.31 | -0.57 | 1.83 | OD | CML504 |
|  | *Gy8* | 8 | 123.0 | pza00739.1-pza01049.1 | 105.79 - 129.04 | 2.61 | 6.21 | 0.25 | -0.49 | 1.97 | OD | CML440 |
|  | *Gy9* | 9 | 50.0 | zhd1.1-pza01999.3 | 22.04 - 23.21 | 4.08 | 7.00 | 0.45 | -0.02 | 0.05 | A | CML440 |
|  | *Asi1* | 1 | 653.0 | phm1438.34-pza03578.1 | 212.39 - 252.22 | 3.14 | 12.87 | 0.30 | -0.40 | 1.34 | D | CML440 |
|  | *Asi2* | 2 | 124.0 | pza01336.1-phm4880.179 | 31.39 - 103.49 | 2.70 | 9.12 | -0.34 | -0.12 | 0.46 | PD | CML504 |
|  | *Asi9* | 9 | 90.0 | pza00947.1-pzb01899.1 | 96.89 - 98.51 | 3.72 | 6.44 | 0.32 | -0.16 | 0.49 | PD | CML440 |
|  |  |  |  |  |  |  |  |  |  |  |  |  |
| ZWW | *Gy2* | 2 | 118.0 | pza01755.1-pza01336.1 | 25.23-31.39 | 3.04 | 5.07 | 0.56 | -0.32 | 0.57 | PD | CML440 |
|  | *Gy6* | 6 | 173.0 | pzb01222.1-pza02815.25 | 164.41 - 167.88 | 3.78 | 5.34 | 0.37 | -0.69 | 1.86 | OD | CML440 |
|  | *Gy8* | 8 | 137.0 | pza00118.5-pza01049.1 | 126.15 - 129.04 | 2.66 | 5.42 | 0.62 | -0.08 | 0.13 | A | CML440 |
|  | *Gy9* | 9 | 84.0 | pza00947.1-pzb01899.1 | 96.88 - 98.50 | 5.01 | 9.32 | -0.81 | 0.16 | 0.19 | A | CML504 |
|  | *Gy10* | 10 | 139.0 | pza01141.1-phm3844.14 | 120.54 - 146.55 | 3.23 | 9.90 | -0.39 | -1.05 | 2.66 | OD | CML504 |
|  | *Asi1a* | 1 | 136.0 | pza03521.1-pza00887.1 | 10.068 - 10.934 | 2.58 | 4.94 | -0.20 | 0.23 | 1.15 | OD | CML504 |
|  | *Asi7* | 7 | 163.0 | pza01542.1-pza02449.13 | 129.79 - 134.84 | 3.72 | 10.31 | 0.09 | 0.51 | 5.63 | OD | CML440 |
|  |  |  |  |  |  |  |  |  |  |  |  |  |
| MWS10 | *Gy1* | 1 | 260.0 | pza03189.4-pza01267.3 | 64.26 - 76.05 | 3.79 | 10.37 | 0.10 | -0.50 | 4.85 | OD | CML440 |
|  | *Gy4* | 4 | 160.0 | pza02779.1-phm1684.20 | 207.11 - 209.04 | 2.68 | 1.42 | 0.04 | 0.18 | 4.34 | OD | CML440 |
|  | *Gy5* | 5 | 178.0 | pza01530.1-pza02408.2 | 37.79 - 189.41 | 2.95 | 2.39 | -0.17 | -0.07 | 0.40 | PD | CML504 |
|  | *Gy6* | 6 | 172.0 | pzb01222.1-pza02815.25 | 164.41 - 167.88 | 4.75 | 8.83 | 0.18 | -0.41 | 2.27 | OD | CML440 |
|  | *Gy7* | 7 | 164.0 | pza01542.1-pza02449.13 | 129.79-138.55 | 2.82 | 1.41 | -0.05 | -0.19 | 3.8 | OD | CML504 |
|  | *Gy8* | 8 | 211.0 | phm1834.47-phm4560.54 | 162.44 - 163.53 | 2.84 | 5.90 | 0.05 | -0.39 | 8.28 | OD | CML440 |
|  | *Gy10* | 10 | 137.0 | pza01141.1-phm3844.14 | 120.53 - 146.55 | 2.86 | 9.56 | -0.14 | 0.44 | 3.14 | OD | CML504 |
|  | *Asi1* | 1 | 138.0 | pza00887.1-pza03521.1 | 10.07 - 10.93 | 4.45 | 7.73 | 0.41 | -0.34 | 0.83 | D | CML440 |
|  | *Asi2* | 2 | 97.0 | pza00590.1-pza01755.1 | 21.99 - 25.23 | 3.21 | 4.87 | 0.37 | -0.13 | 0.35 | PD | CML440 |
|  | *Asi3* | 3 | 148.0 | phm2290.12-phm15449.10 | 121.88 - 125.23 | 2.94 | 3.26 | -0.10 | 0.37 | 3.7 | OD | CML504 |
|  | *Asi5* | 5 | 65.0 | pza01570.1-pza03092.7 | 3.53 - 11.99 | 2.97 | 6.99 | 0.26 | -0.47 | 1.84 | OD | CML440 |
|  |  |  |  |  |  |  |  |  |  |  |  |  |
| MWS11 | *Gy2a* | 2 | 34.0 | phm4425.25-phm6111.5 | 19.84-21.99 | 5.56 | 8.13 | 0.41 | 0.02 | 0.04 | A | CML440 |
|  | *Gy2b* | 2 | 111.0 | pza01755.1-pza01336.1 | 25.23-31.39 | 2.76 | 4.80 | 0.26 | -0.15 | 0.57 | PD | CML440 |
|  | *Gy3* | 3 | 209.0 | pza01962.12-pza03458.1 | 178.23-203.32 | 3.22 | 5.14 | 0.31 | -0.06 | 0.21 | A | CML440 |
|  | *Gy4* | 4 | 229.0 | phm5599.20-pza03322.5 | 239.24-242.02 | 4.25 | 4.07 | 0.22 | -0.17 | 0.79 | PD | CML440 |
|  | *Gy6* | 6 | 170.0 | pzb01222.1-pza02815.25 | 164.41 - 167.88 | 2.55 | 2.53 | 0.12 | -0.23 | 1.91 | OD | CML440 |
|  | *Gy7* | 7 | 164.0 | pza01542.1-pza02449.13 | 129.79-138.55 | 2.91 | 1.52 | -0.01 | -0.24 | 24.0 | OD | CML504 |
|  | *Gy8* | 8 | 143.0 | pza00118.5-phm4203.11 | 126.16-133.53 | 3.27 | 5.59 | -0.16 | -0.43 | 2.71 | OD | CML504 |
|  | *Gy9* | 9 | 150.0 | pza02235.14-pza00708.3 | 132.12-147.38 | 4.81 | 13.76 | 0.45 | -0.19 | 0.41 | PD | CML440 |
|  | *Asi2* | 2 | 69.0 | phm6111.5-pza00590.1 | 21.99-29.99 | 4.05 | 4.28 | 0.15 | -0.59 | 3.94 | OD | CML440 |
|  | *Asi4* | 4 | 57.0 | pza03385.1-phm14717.2 | 37.07-40.52 | 3.16 | 3.61 | -0.36 | -0.24 | 0.66 | PD | CML504 |
|  | *Asi5a* | 5 | 242.0 | pza00963.3-phm3512.186 | 203.43-207.27 | 3.33 | 4.46 | -0.14 | 0.58 | 4.31 | OD | CML504 |
|  | *Asi5b* | 5 | 308.0 | pza01680.3-pza02480.1 | 208.90-214.95 | 4.15 | 8.14 | -0.35 | -0.66 | 1.88 | OD | CML504 |
|  | *Asi7* | 7 | 164.0 | pza01542.1-pza02449.13 | 129.79-134.85 | 3.63 | 4.48 | 0.15 | 0.64 | 4.15 | OD | CML440 |
|  | *Asi8* | 8 | 74.0 | pza01079.1-phm2350.17 | 14.12-23.99 | 5.15 | 7.18 | 0.64 | -0.10 | 0.16 | A | CML440 |
|  | *Asi9a* | 9 | 51.0 | zhd1.1-pza01999.3 | 22.04-23.22 | 7.13 | 9.14 | 0.08 | -0.90 | 10.64 | OD | CML440 |
|  | *Asi9b* | 9 | 118.0 | pza02397.1-phm4905.6 | 133.92-133.92 | 5.28 | 6.59 | -0.63 | 0.06 | 0.10 | A | CML504 |
|  |  |  |  |  |  |  |  |  |  |  |  |  |
| KWS | *Gy1* | 1 | 260.0 | pza03189.4-pza01267.3 | 64.26 - 76.05 | 3.15 | 6.21 | -0.21 | 0.03 | 0.15 | A | CML504 |
|  | *Gy1* | 1 | 465.0 | pza00343.31-phm4752.14 | 294.64 - 298.87 | 5.74 | 12.12 | 0.04 | -0.42 | 10.65 | OD | CML440 |
|  | *Gy4* | 4 | 71.0 | pza02289.2-pza00941.2 | 180.31 - 185.56 | 3.92 | 5.83 | -0.21 | -0.02 | 0.08 | A | CML504 |
|  | *Gy7* | 7 | 164.0 | pza01542.1-pza02449.13 | 129.79-138.55 | 3.06 | 1.57 | -0.04 | 0.17 | 4.25 | OD | CLM504 |
|  | *Gy8* | 8 | 191.0 | phm4757.14-phm1834.47 | 151.45 - 162.44 | 5.35 | 10.96 | 0.03 | 0.40 | 12.28 | OD | CML440 |
|  | *Gy10* | 10 | 140.0 | pza01141.1-phm3844.14 | 120.53 - 146.55 | 6.51 | 15.86 | -0.27 | 0.25 | 0.75 | D | CML504 |
|  | *Asi1* | 1 | 661.0 | phm1438.34-pza03578.1 | 212.39 - ** | 4.16 | 11.14 | -0.49 | 0.13 | 0.26 | PD | CML504 |
|  | *Asi8* | 8 | 52.0 | phm2487.6-pza01079.1 | 8.23 - 14.12 | 4.23 | 7.86 | 0.09 | -0.52 | 5.61 | OD | CML440 |
|  | *Asi9* | 9 | 9.0 | pza01386.3-phm5181.10 | 12.21 - 15.58 | 2.92 | 4.10 | 0.25 | -0.23 | 0.94 | D | CML440 |
|  |  |  |  |  |  |  |  |  |  |  |  |  |

^1^Name of QTLs (*Gy* for grain yield and *Asi* for anthesis-silking interval) followed by a chromosome number. ^2^ Physical position of flanking markers of the QTL in Mb (10^6^ bp). ^3^Predominant genetic effect of a QTL is indicated by A: additive and D: dominant. QTLs with additive effect with positive values were contributed by the parent CML440 and QTL with negative values are from parent CML504. ^4^Gene action determined on the basis of the level of dominance calculated by the ratio between dominant and additive effects of the QTLs (|d/a|) using Stuber et al. (1987) criterion: additive (A) = 0 – 0.20; partial dominance (PD) = 0.21 – 0.80; dominance (D) = 0.81 – 1.20, and overdominance OD > 1.20. ** unknown physical position.

**Table S4.** QTLs and their genomic position, LOD values, genetic effects, gene action and phenotypic variation explained (R^2^) for GY and ASI QTLs mapped in different environments using F_2:3_ families from CML444xCML441

| **Env** |  | **QTL Position** | | | |  |  | **Genetic Effect**^3^ | | | **Gene Action**^4^ | |
| --- | --- | --- | --- | --- | --- | --- | --- | --- | --- | --- | --- | --- |
|  | **QTL**^1^ | **Chr** | **Pos (cM)** | **Marker Interval** | **Physical positon** ^2^ | **LOD** | **R^2^ (%)** | **Add** | **Dom** | **\|d/a\|** | **Nature** | **Direction** |
| MWW | *Gy1a* | 1 | 144.0 | pza03578.1-d8.2 | 252.22-265.20 | 2.93 | 1.47 | -0.24 | 0.08 | 0.34 | PD | CML441 |
|  | *Gy1b* | 1 | 487.0 | pza03183.5-pza03189.4 | 46.07-64.26 | 2.66 | 2.09 | 0.10 | -0.46 | 4.62 | OD | CML444 |
|  | *Gy2* | 2 | 62.0 | pza01280.2-phm3668.12 | 149.43-195.56 | 2.78 | 3.22 | 0.33 | -0.32 | 0.97 | D | CML444 |
|  | *Gy3* | 3 | 227.0 | pza00279.2-pza00279.2 | 52.80-210.16 | 13.27 | 23.69 | 0.98 | 0.48 | 0.49 | PD | CML444 |
|  | *Gy5* | 5 | 363.0 | phm5798.39-pza01304.1 | 71.10-178.58 | 3.40 | 1.63 | -0.21 | 0.15 | 0.74 | PD | CML441 |
|  | *Gy6* | 6 | 50.0 | pza00355.2-phm2551.31 | 78.76-85.13 | 2.51 | 3.19 | 0.22 | -0.47 | 2.13 | OD | CML444 |
|  | *Gy10* | 10 | 318.0 | phm15868.56-pza02527.2 | 137.13-148.49 | 3.00 | 6.56 | 0.52 | 0.41 | 0.78 | PD | CML444 |
|  | *Asi1a* | 1 | 145.0 | pza03578.1-d8.2 | 252.22-265.19 | 2.57 | 1.85 | -0.10 | 0.18 | 1.80 | OD | CML441 |
|  | *Asi1b* | 1 | 571.0 | phm595.30-pza02087.2 | 281.82-284.06 | 3.28 | 4.94 | -0.15 | -0.16 | 1.05 | D | CML441 |
|  | *Asi3* | 3 | 114.0 | phm2423.33-pza00297.2 | 39.99-227.68 | 4.62 | 7.95 | -0.21 | -0.29 | 1.38 | OD | CML441 |
|  | *Asi4* | 4 | 280.0 | pza02027.1-pza03459.1 | 132.98-134.29 | 2.66 | 6.27 | -0.15 | -0.32 | 2.14 | OD | CML441 |
|  | *Asi5* | 5 | 165.0 | pza00963.3-pza02015.11 | 207.27-207.46 | 2.87 | 4.33 | -0.19 | 0.00 | 0.01 | A | CML441 |
|  | *Asi7* | 7 | 63.0 | pza01909.2-pza01210.1 | 6.44-75.09 | 3.38 | 3.30 | -0.15 | 0.17 | 1.13 | D | CML441 |
|  | *Asi10* | 10 | 96.0 | pza01001.2-phm3736.11 | 146.54-147.76 | 3.38 | 9.20 | -0.26 | -0.19 | 0.73 | PD | CML441 |
|  |  |  |  |  |  |  |  |  |  |  |  |  |
| ZWW | *Gy1* | 1 | 306.0 | phm5622.21pza02467.10 | 183.83-196.93 | 4.02 | 12.96 | 0.63 | 0.20 | 0.31 | PD | CML444 |
|  | *Gy2* | 2 | 234.0 | pza00365.2-pza02337.4 | 1.22-15.51 | 2.92 | 7.96 | 0.37 | 0.70 | 1.90 | OD | CML444 |
|  | *Asi1* | 1 | 195.0 | phm3034.3-pza01921.19 | 255.55-261.32 | 3.05 | 11.49 | -0.28 | -0.48 | 1.70 | OD | CML441 |
|  |  |  |  |  |  |  |  |  |  |  |  |  |
| MWS10 | *Gy1a* | 1 | 324.0 | pza03200.2-phm5622.21 | 148.69-183.83 | 6.18 | 9.51 | 0.50 | 0.10 | 0.20 | A | CML444 |
|  | *Gy1b* | 1 | 486.0 | pza03183.5-pza03189.4 | 46.06-64.26 | 2.81 | 2.66 | 0.12 | -0.41 | 3.43 | OD | CML444 |
|  | *Gy2* | 2 | 164.0 | pza02264.5-pzb00901.4 | 3.17-9.41 | 3.89 | 5.71 | 0.43 | -0.35 | 0.81 | PD | CML444 |
|  | *Gy3a* | 3 | 117.0 | pza00297.2-pza03070.9 | 39.99-43.86 | 2.93 | 1.21 | 0.00 | 0.27 | ∞ | OD | CML444 |
|  | *Gy3b* | 3 | 278.0 | pza01154.1-phm2672.19 | 216.03-219.86 | 3.69 | 4.95 | 0.34 | -0.38 | 1.11 | D | CML444 |
|  | *Gy4* | 4 | 85.0 | phm4117.14-phm5780.13 | 215.39-237.58 | 8.27 | 12.62 | 0.58 | 0.14 | 0.24 | PD | CML444 |
|  | *Gy5a* | 5 | 62.0 | pza02480.1-pza02769.1 | 214.95-215.51 | 3.15 | 3.53 | 0.17 | 0.35 | 2.04 | OD | CML444 |
|  | *Gy5b* | 5 | 363.0 | phm5798.39-pza01304.1 | 71.10-178.58 | 3.13 | 1.92 | 0.00 | 0.38 | ∞ | OD | CML444 |
|  | *Gy10* | 10 | 270.0 | phm5740.9-pzb01301.5 | 8.77-9.75 | 3.77 | 4.72 | -0.17 | 0.42 | 2.49 | OD | CML441 |
|  | *Asi1* | 1 | 573.0 | phm595.30-pza02087.2 | 281.82-284.06 | 5.10 | 8.10 | -0.49 | -0.10 | 0.20 | A | CML441 |
|  | *Asi2* | 2 | 262.0 | pza01232.1-pza02939.10 | 155.87-157.15 | 6.10 | 10.13 | -0.50 | 0.16 | 0.33 | PD | CML441 |
|  | *Asi3* | 3 | 114.0 | phm2423.33-pza00297.2 | 39.99-227.68 | 3.08 | 2.08 | -0.23 | -0.27 | 1.17 | D | CML441 |
|  | *Asi5* | 5 | 370.0 | pza02207.1-pza01304.1 | 49.20-178.58 | 3.40 | 3.42 | -0.13 | -0.51 | 3.99 | OD | CML441 |
|  | *Asi6* | 6 | 98.0 | pza00214.1-phm12794.47 | 91.70-128.48 | 2.53 | 3.72 | 0.22 | -0.46 | 2.09 | OD | CML444 |
|  | *Asi10a* | 10 | 150.0 | pza01456.2-phm3844.14 | 135.93-146.55 | 3.22 | 6.82 | 0.06 | -0.66 | 11.72 | OD | CML444 |
|  | *Asi10b* | 10 | 318.0 | phm15868.56-pza02527.2 | 137.13-148.49 | 3.29 | 5.39 | -0.28 | -0.60 | 2.13 | OD | CML441 |
|  |  |  |  |  |  |  |  |  |  |  |  |  |
| MWS11 | *Gy1a* | 1 | 44.0 | pzb01227.6-pza00623.3 | 288.44-293.63 | 2.92 | 2.46 | 0.18 | 0.19 | 1.05 | D | CML444 |
|  | *Gy1b* | 1 | 338.0 | pza03200.2-pza02741.1 | 148.64-161.07 | 2.95 | 3.41 | 0.28 | 0.00 | 0.00 | A | CML444 |
|  | *Gy2* | 2 | 67.0 | pza01280.2-phm3668.12 | 149.43-195.56 | 2.73 | 3.58 | 0.13 | -0.37 | 2.83 | OD | CML444 |
|  | *Gy3a* | 3 | 113.0 | pza00297.2-pza03070.9 | 39.99-43.86 | 6.07 | 10.32 | 0.11 | 0.73 | 6.97 | OD | CML444 |
|  | *Gy3b* | 3 | 221.0 | pza00279.2-pza02616.1 | 52.80-210.16 | 11.02 | 19.02 | 0.58 | 0.31 | 0.54 | PD | CML444 |
|  | *Gy5* | 5 | 63.0 | pza02480.1-pza02769.1 | 214.95-215.51 | 3.13 | 3.26 | 0.28 | -0.03 | 0.10 | A | CML444 |
|  | *Gy10* | 10 | 271.0 | phm5740.9-pzb01301.5 | 8.77-9.75 | 2.86 | 2.55 | -0.24 | 0.04 | 0.34 | PD | CML441 |
|  | *Asi1a* | 1 | 48.0 | pzb01227.6-pza00623.3 | 288.44-293.63 | 2.85 | 2.45 | 0.18 | 0.20 | 1.13 | D | CML441 |
|  | *Asi1b* | 1 | 491.0 | pza03183.5-pza03189.4 | 46.06-64.26 | 3.01 | 3.34 | 0.14 | 0.45 | 3.09 | OD | CML444 |
|  | *Asi3a* | 3 | 116.0 | phm2423.33-pza00297.2 | 39.99-227.68 | 2.88 | 0.97 | -0.19 | 0.20 | 1.05 | D | CML441 |
|  | *Asi3b* | 3 | 274.0 | pza01154.1-phm2672.19 | 216.03-219.86 | 2.56 | 2.55 | -0.12 | 0.48 | 4.04 | OD | CML441 |
|  | *Asi6* | 6 | 98.0 | pza00214.1-phm12794.47 | 91.70-128.48 | 2.72 | 2.85 | 0.37 | -0.11 | 0.30 | PD | CML444 |
|  | *Asi7* | 7 | 62.0 | pza01909.2-pza01210.1 | 6.44-75.09 | 2.65 | 0.98 | -0.24 | 0.06 | 0.20 | A | CML441 |
|  | *Asi10* | 10 | 100.0 | pza01001.2-phm3736.11 | 146.54-147.76 | 2.55 | 3.60 | -0.33 | -0.38 | 1.15 | D | CML441 |
|  |  |  |  |  |  |  | 14.79 |  |  |  |  |  |
| KWS | *Gy1a* | 1 | 47.0 | pzb01227.6-pza00623.3 | 288.44-293.63 | 3.19 | 7.17 | 0.05 | -0.27 | 5.02 | OD | CML444 |
|  | *Gy1b* | 1 | 397.0 | pzb00872.3-pzb01062.3 | 46.25-56.85 | 4.02 | 6.72 | 0.18 | 0.07 | 0.37 | PD | CML444 |
|  | *Gy2* | 2 | 138.0 | pza02727.1-phm482.27 | 11.10-227.92 | 3.11 | 5.73 | -0.49 | 0.47 | 0.97 | D | CML441 |
|  | *Gy3a* | 3 | 115.0 | pza00297.2-pza03070.9 | 39.99-43.86 | 2.86 | 2.16 | 0.00 | -0.41 | ∞ | OD | CML441 |
|  | *Gy4* | 4 | 302.0 | fea2.3-pza02194.1 | 132.74-180.31 | 2.88 | 4.21 | 0.15 | 0.06 | 0.37 | PD | CML444 |
|  | *Gy9* | 9 | 38.0 | pzb01110.6-pza01062.1 | 88.06-24.03 | 5.57 | 12.90 | 0.27 | -0.01 | 0.03 | A | CML444 |
|  | *Gy10* | 10 | 319.0 | phm15868.56-pza02527.2 | 137.13-148.49 | 3.43 | 6.49 | -0.02 | -0.28 | 15.70 | OD | CML441 |
|  | *Asi1* | 1 | 398.0 | pzb00872.3-pzb01062.3 | 46.26-56.85 | 4.95 | 7.21 | -0.93 | -0.26 | 0.28 | PD | CML441 |
|  | *Asi2* | 2 | 134.0 | phm482.27-pza02727.1 | 11.10-227.92 | 4.33 | 6.45 | 0.98 | -0.48 | 0.50 | PD | CML444 |
|  | *Asi3a* | 3 | 118.0 | pza00297.2-pza03070.9 | 39.99-43.86 | 3.46 | 3.09 | -0.38 | 0.90 | 2.38 | OD | CML441 |
|  | *Asi3b* | 3 | 249.0 | pza02516.1-pza03391.1 | 219.86-208.18 | 3.20 | 4.74 | -0.25 | -0.93 | 3.70 | OD | CML441 |
|  | *Asi7* | 7 | 69.0 | pza01909.2-pza01210.1 | 6.44-75.09 | 3.48 | 4.28 | -0.76 | 0.04 | 0.05 | A | CML441 |
|  | *Asi9a* | 9 | 37.0 | pzb01110.6-pza01062.1 | 24.03-88.06 | 2.67 | 3.06 | -0.50 | 0.39 | 0.78 | PD | CML441 |
|  | *Asi9b* | 9 | 177.0 | phm816.29-pza01715.2 | 142.05-142.95 | 4.63 | 6.76 | -0.53 | -0.88 | 1.65 | OD | CML441 |
|  | *Asi10* | 10 | 267.0 | phm5740.9-pzb01301.5 | 8.77-9.75 | 3.32 | 4.35 | -0.75 | -0.19 | 0.25 | PD | CML441 |
|  |  |  |  |  |  |  |  |  |  |  |  |  |
| ZWS | *Gy1a* | 1 | 217.0 | pza01921.19-pza03064.9 | 261.31-294.41 | 2.61 | 5.77 | 0.14 | 0.16 | 1.14 | D | CML444 |
|  | *Gy1b* | 1 | 527.0 | pza02284.1-phm1653.32 | 9.27-14.89 | 4.60 | 10.22 | 0.01 | 0.36 | 36.0 | OD | CML444 |
|  | *Gy2* | 2 | 234.0 | pza00365.2-pza02337.4 | 1.22-15.51 | 2.51 | 4.70 | 0.12 | 0.22 | 1.91 | OD | CML44 |
|  | *Gy3* | 3 | 119.0 | pza00297.2-pza03070.9 | 39.99-43.86 | 3.38 | 5.25 | 0.03 | -0.24 | 6.91 | OD | CML444 |
|  | *Gy5* | 5 | 367.0 | pza01304.1-pza02207.1 | 49.20-178.58 | 3.17 | 5.25 | 0.15 | 0.17 | 1.13 | D | CML444 |
|  | *Gy7* | 7 | 108.0 | pza00153.7-pza01946.7 | **-123.60 | 3.47 | 9.07 | -0.22 | 0.20 | 0.93 | PD | CML441 |
|  | *Asi1* | 1 | 116.0 | d8.2-pzb00114.1 | 265.20-275.98 | 2.53 | 4.45 | -0.76 | -0.99 | 1.30 | OD | CML441 |
|  | *Asi3* | 3 | 120.0 | pza00297.2-pza03070.9 | 39.99-43.86 | 2.78 | 3.02 | -0.62 | -0.59 | 0.95 | D | CML441 |
|  | *Asi6* | 6 | 127.0 | phm1190.3-phm12794.47 | 120.23-128.48 | 3.26 | 7.33 | 0.75 | 0.83 | 1.10 | D | CML444 |
|  | *Asi7* | 7 | 61.0 | pza01909.2-pza01210.1 | 6.43-75.09 | 3.85 | 12.27 | -1.48 | 0.80 | 0.54 | PD | CML441 |
|  |  |  |  |  |  |  |  |  |  |  |  |  |

^1^Name of QTLs (*Gy* for grain yield and *Asi* for anthesis-silking interval) followed by a chromosome number. ^2^ Physical position of flanking markers of the QTL in Mb (10^6^ bp). ^3^Predominant genetic effect of a QTL is indicated by A: additive and D: dominant. QTLs with additive effect with positive values were contributed by the parent CML444 and QTL with negative values are from parent CML441. ^4^Gene action determined on the basis of the level of dominance calculated by the ratio between dominant and additive effects of the QTLs (|d/a|) using Stuber et al. (1987) criterion: additive (A) = 0 – 0.20; partial dominance (PD) = 0.21 – 0.80; dominance (D) = 0.81 – 1.20, and overdominance OD > 1.20. ** unknown physical position.

**Table S5.** Means of the best and worst families under water stress (WS) and their corresponding values under well watered (WW) environments in three bi-parental maize populations.

| Population | **Best families** | | | **Worst families** | | |
| --- | --- | --- | --- | --- | --- | --- |
|  | Pedigree | WS | WW | Pedigree | WS | WW |
| CML444xMALAWI | CML444/MALAWI-291 | 6.76 | 8.10 | CML444/MALAWI-395 | 2.98 | 6.95 |
|  | CML444/MALAWI-330 | 6.49 | 8.81 | CML444/MALAWI-205 | 2.89 | 10.81 |
|  | CML444/MALAWI-352 | 6.42 | 11.22 | CML444/MALAWI-120 | 2.80 | 10.39 |
|  | CML444/MALAWI-52 | 6.41 | 10.74 | CML444/MALAWI-230 | 2.78 | 8.77 |
|  | CML444/MALAWI-49 | 6.31 | 10.02 | CML444/MALAWI-322 | 2.74 | 10.46 |
|  | CML444/MALAWI-342 | 6.27 | 11.45 | CML444/MALAWI-318 | 2.66 | 7.49 |
|  | CML444/MALAWI-377 | 6.22 | 9.18 | CML444/MALAWI-69 | 2.61 | 8.47 |
|  | CML444/MALAWI-327 | 6.20 | 9.56 | CML444/MALAWI-187 | 2.52 | 7.67 |
|  | CML444/MALAWI-50 | 6.08 | 10.73 | CML444/MALAWI-46 | 2.45 | 7.84 |
|  | CML444/MALAWI-117 | 6.07 | 9.17 | CML444/MALAWI-186 | 2.25 | 5.97 |
|  | CML444/MALAWI-356 | 6.03 | 10.23 | CML444/MALAWI-388 | 2.20 | 6.85 |
|  | Mean of lines | 6.30 | 9.93 |  | 2.63 | 8.34 |
|  | Mean of population | 4.64 | 8.58 |  | 4.64 | 8.58 |
| CML440xCML504 | CML440/CML504-B-439 | 6.35 | 9.95 | CML440/COMPE2-B-186 | 4.03 | 7.29 |
|  | CML440/CML504-B-528 | 6.15 | 12.27 | CML440/COMPE2-B-337 | 4.02 | 9.75 |
|  | CML440/CML504-B-551 | 6.10 | 10.12 | CML440/COMPE2-B-429 | 3.95 | 7.79 |
|  | CML440/CML504-B-485 | 6.07 | 11.85 | CML440/COMPE2-B-295 | 3.85 | 7.52 |
|  | CML440/CML504-B-559 | 6.00 | 11.05 | CML440/COMPE2-B-21 | 3.85 | 10.59 |
|  | CML440/CML504-B-814 | 5.98 | 10.37 | CML440/COMPE2-B-458 | 3.78 | 7.99 |
|  | CML440/CML504-B-292 | 5.91 | 9.82 | CML440/COMPE2-B-414 | 3.70 | 10.43 |
|  | CML440/CML504-B-389 | 5.88 | 13.61 | CML440/COMPE2-B-139 | 3.62 | 6.88 |
|  | CML440/CML504-B-597 | 5.83 | 11.60 | CML440/COMPE2-B-469 | 3.55 | 8.59 |
|  | CML440/CML504-B-627 | 5.78 | 14.22 | CML440/COMPE2-B-318 | 3.43 | 9.10 |
|  | CML440/CML504-B-633 | 5.75 | 12.24 | CML440/COMPE2-B-375 | 3.38 | 10.66 |
|  | Mean of lines | 5.98 | 11.55 |  | 3.74 | 8.78 |
|  | Mean of population | 4.88 | 10.00 |  | 4.88 | 10.00 |
| CML444xCML441 | CML441/CML444-B-3 | 5.79 | 10.78 | CML441/CML444-B-337 | 2.60 | 7.32 |
|  | CML441/CML444-B-454 | 5.25 | 11.92 | CML441/CML444-B-735 | 2.58 | 9.12 |
|  | CML441/CML444-B-382 | 5.17 | 9.82 | CML441/CML444-B-106 | 2.56 | 5.97 |
|  | CML441/CML444-B-548 | 5.04 | 10.78 | CML441/CML444-B-615 | 2.52 | 10.16 |
|  | CML441/CML444-B-103 | 5.01 | 10.76 | CML441/CML444-B-744 | 2.49 | 7.84 |
|  | CML441/CML444-B-68 | 4.92 | 11.01 | CML441/CML444-B-813 | 2.45 | 5.77 |
|  | CML441/CML444-B-457 | 4.89 | 11.33 | CML441/CML444-B-698 | 2.38 | 8.19 |
|  | CML441/CML444-B-7 | 4.88 | 12.12 | CML441/CML444-B-550 | 2.29 | 9.24 |
|  | CML441/CML444-B-417 | 4.82 | 11.11 | CML441/CML444-B-246 | 1.80 | 8.47 |
|  | CML441/CML444-B-46 | 4.80 | 9.67 | CML441/CML444-B-690 | 1.38 | 3.27 |
|  | CML441/CML444-B-514 | 4.74 | 11.55 | CML441/CML444-B-728 | 0.00 | 7.14 |
|  | Mean of lines | 5.03 | 10.99 |  | 2.10 | 7.50 |
|  | Mean of population | 3.66 | 9.91 |  | 3.66 | 9.91 |

**Supplementary Reference:**

1. Casagrande EC, Farias JRB, et al.(2001) Expressão gênica diferencial durante déficit hídrico em soja. Rev Bras Fisiol Veg 13: 168-184.
2. Cominelli E, Galbiati M, Vavasseur A, Conti L, Sala T, Vuylsteke M, Leonhardt N, Dellaporta SL, Tonelli C (2005) A guard-cell-specific MYB transcription factor regulates stomatal movements and plant drought tolerance. Curr Biol 15:1196-200. doi:10.1016/j.cub.2005.05.048
3. Gigon A, Matos A-R, Laffray D, Zuily-Fodil Y, Pham-Thi A-T (2004) Effect of drought stress on lipid metabolism in the leaves of *Arabidopsis thaliana* (Ecotype Columbia). Ann Bot 94: 345-351. doi:10.1093/aob/mch150
4. Hu X, Li Y, Li C, Yang H, Wang W, Lu M (2010) Characterization of small heat shock proteins associated with maize tolerance to combined drought and heat stress. J Plant Growth Regul 29:455–464. doi:10.1007/s00344-010-9157-9
5. Pastore D, Trono D, Laus MN, Di Fonzo N, Flagella Z (2007) Possible plant mitochondria involvement in cell adaptation to drought stress - A case study: Durum wheat mitochondria. J Exp Bot 58: 195-210. doi:10.1093/jxb/erl273
6. Raab S, Toth Z, De Groot C, Stamminger T, Hoth S (2006) ABA-responsive RNA-binding proteins are involved in chloroplast and stromule function in Arabidopsis seedlings. Planta 224: 900–914. doi:10.1007/s00425-006-0282-4
7. Yu X, Wang B, Zhang C, Xu W, He J, Zhu L, Wang S (2012) Effect of root restriction on nitrogen levels and glutamine synthetase activity in 'Kyoho' grapevines. Sci Hortic 137: 156-163. doi:10.1016/j.scienta.2012.01.025
8. Zhang X, Liu S, Takano T (2008) Two cysteine proteinase inhibitors from *Arabidopsis thaliana*, AtCYSa and AtCYSb, increasing the salt, drought, oxidation and cold tolerance. Plant Mol Biol 68: 131-143. doi:10.1007/s11103-008-9357-x
